# dMyc suppresses CTG-induced cytotoxicity in the *Drosophila* model of DM1 by reducing autophagy and cell death

**DOI:** 10.1038/s41420-026-03123-w

**Published:** 2026-04-20

**Authors:** Dipti Chakraborty, Naorem Tarundas Singh, Shreya Borthakur, Mayanglambam Dhruba Singh

**Affiliations:** BRIC-National Brain Research Centre, Manesar, India

**Keywords:** Mechanisms of disease, Experimental models of disease

## Abstract

Myotonic Dystrophy Type 1 (DM1) is a complex, genetic, and multisystemic disorder caused by the expansion of CTG trinucleotide repeats in the Dystrophia Myotonica Protein Kinase gene, leading to the formation of toxic RNA foci, which finally result in progressive muscle weakness, myotonia, and systemic complications affecting almost every organ system of the body. Despite its severity and high prevalence, effective therapeutic strategies remain elusive. Our study aims to identify a genetic modifier with therapeutic potential. We used transgenic flies expressing pathogenic CTG250 and CTG270 repeats as a *Drosophila* model of DM1, which recapitulated the hallmark features: formation of RNA foci, muscle fibre degeneration, impaired locomotor activity, and shortened lifespan. *Drosophila* Myc (dMyc), also known as diminutive, is a highly conserved transcription factor that plays a crucial role in cellular growth, metabolism, and autophagy. We found that targeted overexpression of dMyc significantly ameliorated disease phenotypes, including improved muscle integrity, enhanced motor function, extended lifespan, and reduced RNA foci. Our findings also revealed that dMyc expression is significantly reduced in flies with disease caused by abnormal CTG expansion. Overexpression of dMyc led to a marked decrease in autophagy and apoptosis. Our findings highlight impaired dMyc expression in CTG-mediated pathogenesis in the DM1 model and suggest that modulation of Myc expression could be a promising therapeutic intervention for DM1.

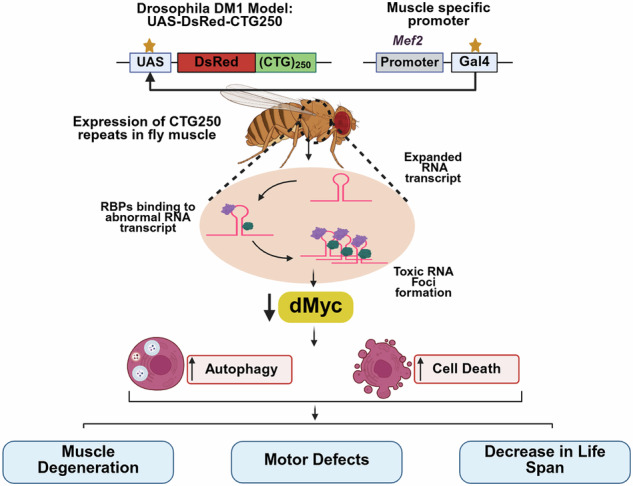

## Introduction

Myotonic dystrophy type 1 (DM1) is an autosomal dominant, multisystemic neuromuscular disorder first described by Hans Gustav Wilhelm Steinert in 1909 [1, 2]. It is the most prevalent and familiar adult-onset muscular dystrophy, characterised by progressive muscle weakness, myotonia, and a multi-system disorder affecting skeletal muscle, the eye, heart, endocrine system, and central nervous system [3, 4]. A recent meta-analysis reported a pooled prevalence estimate of 9.27 cases per 100,000 individuals, ranging from 0.37 to 36.29 per 100,000 depending on geographic and methodological factors [5]. Although DM1 affects multiple organ systems, its most pronounced manifestations are found in skeletal muscle [1].

DM1 is caused by the expansion of a CTG trinucleotide repeat in the 3′ untranslated region (UTR) of the Dystrophia Myotonica Protein Kinase (DMPK) gene [6, 7]. While unaffected individuals typically possess fewer than 30 CTG repeats, affected individuals can carry expansions ranging from approximately 50 to over 2000 repeats. According to the prevailing model of RNA Gain-of-function, these RNA transcripts with expanded CUG repeats form discrete RNA foci that aberrantly sequester RNA-binding proteins, particularly the Muscleblind-like (MBNL) family of splicing regulators [1, 8]. The loss of functional MBNL proteins disrupts normal alternative splicing and other RNA processing events in a tissue-specific manner. This widespread splicing dysregulation across multiple organs that express DMPK underlies the multisystemic nature of DM1 pathology, contributing to its diverse clinical manifestations [9]. Over the past decade, advancements in therapeutics for DM1 have primarily focused on addressing specific clinical symptoms, such as treating cardiac manifestations, diabetes mellitus, and cataracts [10]. However, the molecular mechanism of muscle atrophy in DM1 is, thus, poorly understood. Therefore, recent publications underscore the increasing demand for treatments that address the complex multisystemic nature of DM1 [11]. The current investigation aims to identify novel genetic modifiers that inhibit the cellular toxicity caused by CUG expansion and may serve as targets for developing and evaluating treatment approaches. We utilise the *Drosophila* model of DM1 that expresses 250 or 270 uninterrupted CTG repeats (namely CTG250 or CTG270) in the 3’ untranslated region of red fluorescent protein (DsRed) under the control of UAS [12]. Overexpression of CTG250 or CTG270 leads to reduced eye size, structural abnormalities in muscle tissue, compromised motor performance, and a marked decrease in the lifespan of the flies. We identify dMyc (also known as *diminutive*) as a novel genetic modulator of CUG-induced toxicity in the *Drosophila* DM1 model. dMyc is homologous to the c-Myc proto-oncogene in humans. Like the human Myc protein, dMyc is a transcription factor that regulates various cellular activities, including cell proliferation, growth, and apoptosis, and is evolutionarily conserved across species [13–15]. We observe that targeted overexpression of dMyc significantly improves the muscle morphology, motor ability, and lifespan in DM1 model flies. Our findings reveal that dMyc is downregulated in CTG-expanded flies. Furthermore, dMyc could significantly reduce the formation of toxic RNA foci, aberrant autophagy, and apoptotic cell death. Our findings strongly suggest that dMyc may play a crucial role in the pathogenesis of expanded CTG and is a promising target for combating DM1-associated toxicity and muscle degeneration.

## Results

### dMyc improves the overall health of the DM1 model flies

**Fig. 1:**
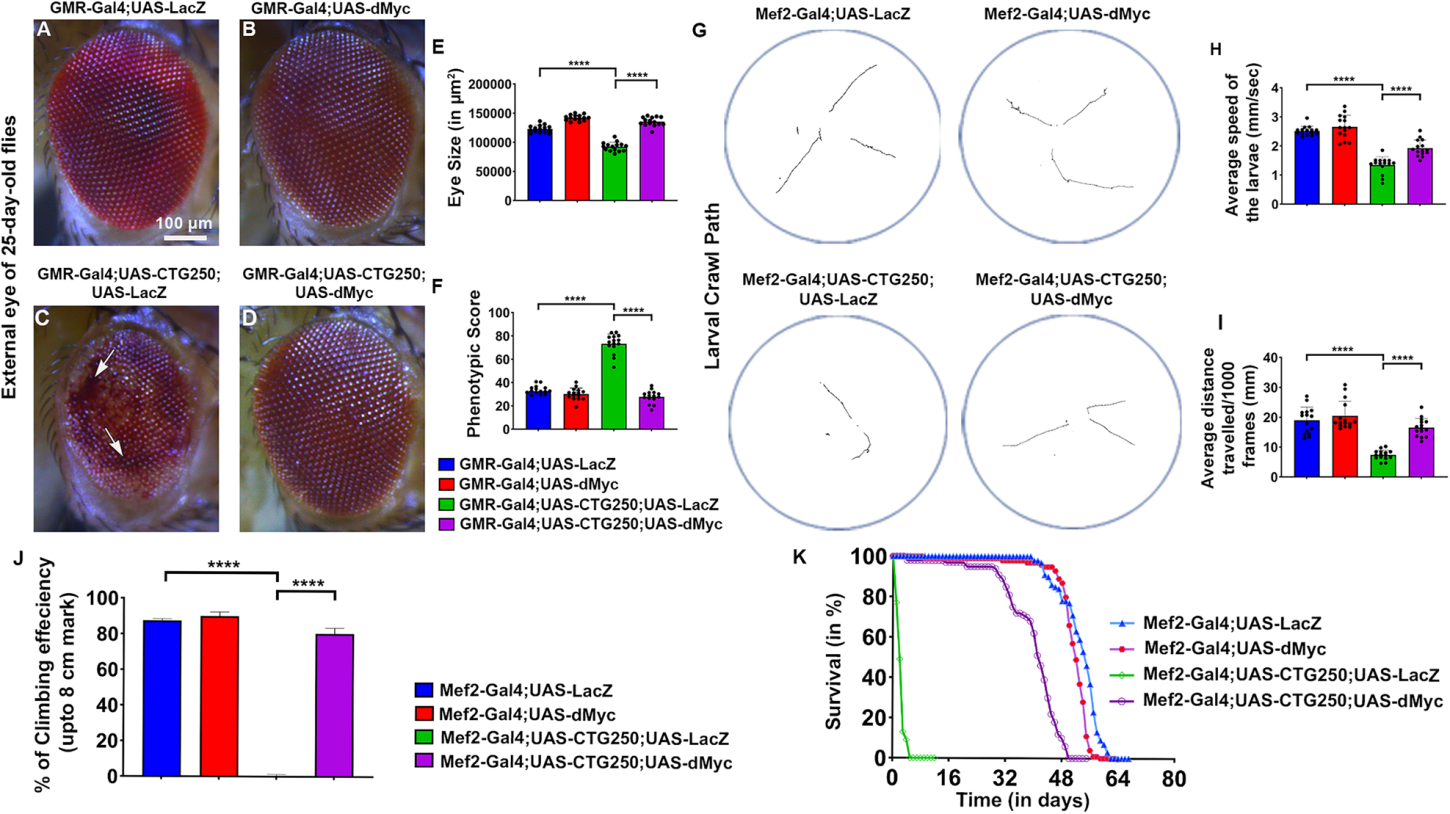
dMyc improved the overall health of the DM1 model flies. **A–D** Bright-field images of the 25-day-old adult female fly eyes (*n* = 15 flies/genotype). **A** Eye image of the control fly. **B** Eye image of the dMyc overexpressed fly. **C** Flies expressing CTG250 repeats displayed a marked reduction in eye size, loss of pigmentation, and formation of necrotic patches in the eye (indicated by the arrow). **D** dMyc overexpression in the CTG250 flies led to a significant improvement in eye size, pigmentation, and roughness of the eye. **E** Bar graph comparing eye size across the genotypes. Quantification data showed a significant decrease in eye size in disease flies. **F** Bar graph comparing the Phenotypic score between different genotypes using Flynotyper (*n* = 15 flies/genotype). **G** Crawling assay path length diagrams were generated to compare the motor ability of the third-instar larvae of different genotypes (*n* = 15 flies/genotype). Diseased larvae exhibited shorter path lengths than controls, whereas dMyc overexpression in the disease background improved path length. **H** Bar graph representing the average speed of crawling larvae of different genotypes. Diseased larvae exhibited a reduced average speed compared to controls, whereas Myc overexpression in the disease background improved speed. **I** Bar graph representing the average distance travelled by different genotypes. Diseased larvae showed a reduced average distance compared to controls, whereas Myc overexpression in the disease background increased the distance travelled. **J** Bar graph representing the climbing ability of the flies (*n* = ~120 flies/genotype). Flies expressing CTG250 repeats had impaired climbing ability compared with controls. Significant improvement in climbing performance was observed in diseased flies expressing dMyc. **K** The lifespan of adult flies across all genotypes was measured by maintaining them at 25 °C (*n* ~ 150 flies/genotype). A drastic lethality rate was observed 2 days post-eclosion in diseased flies, which was significantly shorter than that of control flies. Compared with diseased flies, dMyc-expressing flies in the diseased condition survived for longer, indicating a significant restoration of lifespan and validating a protective role of dMyc in mitigating CTG-associated muscle toxicity. Survival curves were generated by the Kaplan-Meier method. Statistical analyses were performed using the log-rank test. All bar graphs were presented as mean ± SD and analysed using one-way ANOVA with Tukey’s post-hoc analysis across genotypes. Statistical significance was indicated as follows: ****P < 0.0001. Scale bar (**A–D**): 100 µm.

We utilised a transgenic *Drosophila* model of DM1, featuring uninterrupted CTG250 or CTG270 repeats inserted into the 3′ UTR of a DsRed reporter gene [12]. Expression of this expanded CTG repeat in the *Drosophila* eye using the GMR-Gal4 driver exhibited a robust neurodegenerative phenotype by 25 days post-eclosion, evident from notable loss of pigmentation, reduction in size, and formation of necrotic patches in both the CTG250 and CTG270 flies (Figs. 1A, C, E and S1A, C, E). To identify a novel genetic modifier that could suppress CTG-mediated pathogenesis, a genetic screen was conducted using the *Drosophila* eye as a readout of the potential phenotypes. We observed that dMyc overexpression in the disease background significantly improved eye morphology, reduced the roughness of the external eye surface, increased eye size, and improved pigmentation (Figs. 1D, E and S1D, E). DM1 model flies showed a significantly higher phenotypic score in Flynotyper, which measured the disorderliness of ommatidial arrangement in the eye (Figs. 1F and S1F) [16]. Interestingly, DM1 model flies with dMyc showed a significant reduction in the phenotypic score, comparable to the control flies (Figs. 1F and S1F). These observations provide preliminary insight into the potential role of dMyc in DM1 pathogenesis.

DM1 is a form of muscular dystrophy that primarily affects patients’ muscles. Therefore, we investigated whether dMyc could suppress disease pathogenesis in *Drosophila* muscle. Using a muscle-specific Mef2-Gal4 driver, CTG250 repeats were expressed in the muscle tissues of the flies. First, we tested the crawling behaviour of third-instar larvae (Figs. 1G, S2 and Videos 1–4). We observed a marked reduction in the average speed and distance travelled by diseased larvae compared to controls, and a significant improvement in the dMyc-overexpressed disease larvae (Fig. 1G, H, I and Table 1). Next, we conducted a negative geotaxis assay to investigate whether the enhanced locomotion observed in larvae is reflected in the adult flies (Fig. S3 and Video 5). Disease flies expressing CTG250 showed impaired climbing ability, with only 1.9% reaching the 8 cm mark (Fig. 1J). In contrast, a significant improvement in climbing was observed in the disease flies expressing dMyc (Fig. 1J). This indicated that overexpression of dMyc reduced CTG250-mediated toxicity and conferred functional rescue in both larval and adult stages. Furthermore, we investigated whether dMyc could also extend the lifespan of CTG250 flies. Therefore, we performed a longevity assay. A drastic lethality rate was observed 2 days post-eclosion in disease flies, which was markedly reduced in disease flies expressing dMyc, indicating a significant restoration of their health and validating a protective role of dMyc in mitigating CTG-associated muscle toxicity (Fig. 1K and Table 2).

**Table 1:** dMyc improves the average speed of CTG250-expressing flies.

Genotypes	Average speed (mm/second)
Mef2-Gal4;UAS-LacZ	2.52 ± 0.15
Mef2-Gal4;UAS-dMyc	2.67 ± 0.38
Mef2-Gal4;UAS-CTG250;UAS-LacZ	1.33 ± 0.27
Mef2-Gal4;UAS-CTG250;UAS-dMyc	1.95 ± 0.27

**Table 2:** dMyc improves the average life span in CTG250 flies.

Genotypes	Average life span (mean ± SD)
Mef2-Gal4;UAS-LacZ	53.56 ± 0.49 days
Mef2-Gal4;UAS-dMyc	51.52 ± 0.75 days
Mef2-Gal4;UAS-CTG250;UAS-LacZ	2.5 ± 0.1 days
Mef2-Gal4;UAS-CTG250;UAS-dMyc	40.07 ± 0.75 days

### dMyc improves the muscle morphology of the adult CTG250 flies

**Fig. 2:**
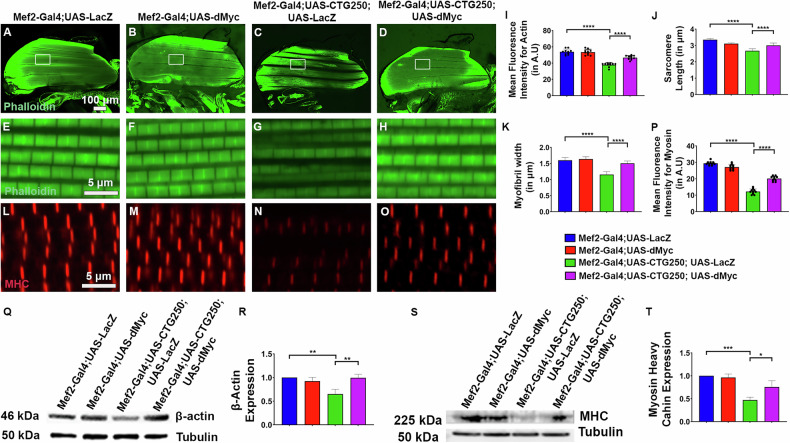
Overexpression of dMyc improved the muscle architecture of the *Drosophila* Dorso-lateral flight muscles (DLMs). **A–D** Hemithoraces of 3-day-old female flies with different genotypes under the control of the Mef2-Gal4, exposing the DLMs of the indirect flight muscles (IFMs), and imaged using confocal microscopy (*n* = 10 flies/genotype). Actin filaments were stained with phalloidin and visualised in green to assess muscle structure. **A**, **B** Proper arrangement of the actin filaments with clear and distinct DLMs, in the control flies, and dMyc overexpressed flies. **C** Diseased flies had atrophied DLMs. **D** dMyc overexpression improved the architecture of the DLMs of the diseased flies. **E–H** 100X with 8X zoomed images of the IFMs of the flies showing the sarcomeres (area of the white box in (**A**–**D**)). **E**, **F** In control flies, sarcomeres were structurally regular. **G** Disorganisation of actin filaments affected the structure of the sarcomeres in diseased flies. **H** dMyc in the disease background improved and restored the F-actin strands, contributing to improved muscle integrity and sarcomere structure. **I** Bar graph comparing the mean fluorescence intensity of the actin filaments across the genotypes. **J** Bar graph comparing the length of sarcomeres of the IFM across the genotypes. **K** Bar graph comparing the myofibril width between the genotypes, where diseased flies exhibited thinner F-actin strands, indicative of disrupted muscle architecture. **L–O** Hemithoraces of all genotypes (*n* = 10 flies/genotype). 100X with 8X zoomed images of the IFMs of the flies showing the M-lines using anti-MHC antibody. **L**, **M** In control and dMyc flies, M-lines were distinct. **N** The thinner and less intensely stained M-lines were observed in the diseased flies. **O** dMyc in the disease background improved M-lines, contributing to improved overall muscle integrity. **P** Bar graph showing the quantification of myosin band showing mean fluorescence intensity across the genotypes. **Q** Western blot results showed a significant reduction in β-actin expression in the disease flies, which was improved in the rescue flies (*n* = 40 flies/genotype, *N* = 3). **R** Graph representing quantification of β-actin immunoblots across the genotypes. **S** Western blot showing reduced myosin heavy chain protein expression in disease flies, which was significantly improved in rescue flies (*n* = 40 flies/genotype, *N* = 3). **T** Graph showing quantification of myosin heavy chain immunoblots across the genotypes. All bar graphs were presented as mean ± SD and analysed using one-way ANOVA with Tukey’s post-hoc analysis across genotypes. Statistical significance was indicated as follows: *P < 0.05, **P < 0.01, ***P < 0.001, ****P < 0.0001. Green: Phalloidin (**A**–**H**), Red: MHC (**L**–**O**). Scale bar (**A**–**D**): 100 µm, (**E**–**H,** **L**–**O**): 5 µm.

To address the effect of expanded CTG on sarcomere organisation and to determine whether the motor improvement conferred by dMyc correlated with improvements in muscle architecture in the rescue flies, we analysed muscle morphology. We examined the arrangement of F-actin in the Dorsal Longitudinal Muscles (DLMs) of the Indirect Flight Muscles (IFMs) using phalloidin staining in 3-day-old flies. The muscle fibres in control and dMyc-overexpressed flies exhibited a regular F-actin arrangement (Fig. 2A, B, E, F). However, the DLMs of the CTG250 flies were atrophied (Fig. 2C). Detailed analysis of the actin cytoskeleton revealed a striking change in fibre organisation (Fig. 2G). dMyc in the disease background improved the organisation of DLMs (Fig. 2D, H). In addition, diseased flies showed reduced F-actin staining, with a Mean Fluorescence Intensity significantly lower than that of the control flies, and this was improved in the rescue flies (Fig. 2I). We also observed significant changes in the myofibrils upon examining the phalloidin-stained F-actin of the sarcomere. The control flies had a typical regular sarcomere organisation (Fig. 2E, J, K and Table 3). However, the sarcomeres of the disease flies exhibited substantial structural alterations, which were significantly shorter than those of the control flies (Fig. 2G, J, K and Table 3). Disease flies with dMyc overexpression showed significantly increased mean sarcomere length and width (Fig. 2H, J, K and Table 3). Given the close association between myosin and actin, we also examined the distribution pattern of Myosin Heavy Chain (MHC) in the IFMs. Specifically, the DLMs of the hemithoraces were stained for MHC to visualise the muscle fibrils (Fig. S4). In the control and dMyc-overexpressing flies, high-magnification images revealed that the M-lines were distinctive, well-defined, and exhibited a regular, alternating pattern of myosin band arrangement (Fig. 2L, M). But, in the disease flies, M-lines were barely detectable and appeared considerably thinner. The M-line was disrupted, with a diffuse, faint band, indicating muscle fibre degeneration (Fig. 2N, P). However, the diffuse MHC distribution pattern in CTG250 flies was rescued in dMyc-overexpressed disease flies (Fig. 2O, P). The change in MHC distribution in the disease flies might be an indirect consequence of disrupted actin organisation. Furthermore, our western blot result also showed a marked reduction in actin and myosin heavy chain protein levels in the disease flies, which was significantly improved in the rescue flies (Fig. 2Q–T). These findings indicated that dMyc overexpression restored actin and Myosin cytoskeletal organisation, which likely contributed to the structural rescue of IFMs in DM1 flies. This improvement was consistently observed across multiple structural levels of the IFMs, including overall muscle morphology, myofibrillar arrangement, and sarcomere organisation.

**Table 3:** The mean length and width of the sarcomere are increased by dMyc overexpression.

Genotypes	Mean length of sarcomere (mean ± SD)	Mean width of sarcomere (mean ± SD)
Mef2-Gal4;UAS-LacZ	3.35 ± 0.08 μm	1.59 ± 0.08 μm
Mef2-Gal4;UAS-dMyc	3.10 ± 0.04 μm	1.63 ± 0.07 μm
Mef2-Gal4;UAS-CTG250;UAS-LacZ	2.67 ± 0.12 μm	1.15 ± 0.09 μm
Mef2-Gal4;UAS-CTG250;UAS-dMyc	3.00 ± 0.14 μm	1.50 ± 0.07 μm

### dMyc expression is impaired by expanded CTG, and its overexpression reduces RNA Foci

**Fig. 3:**
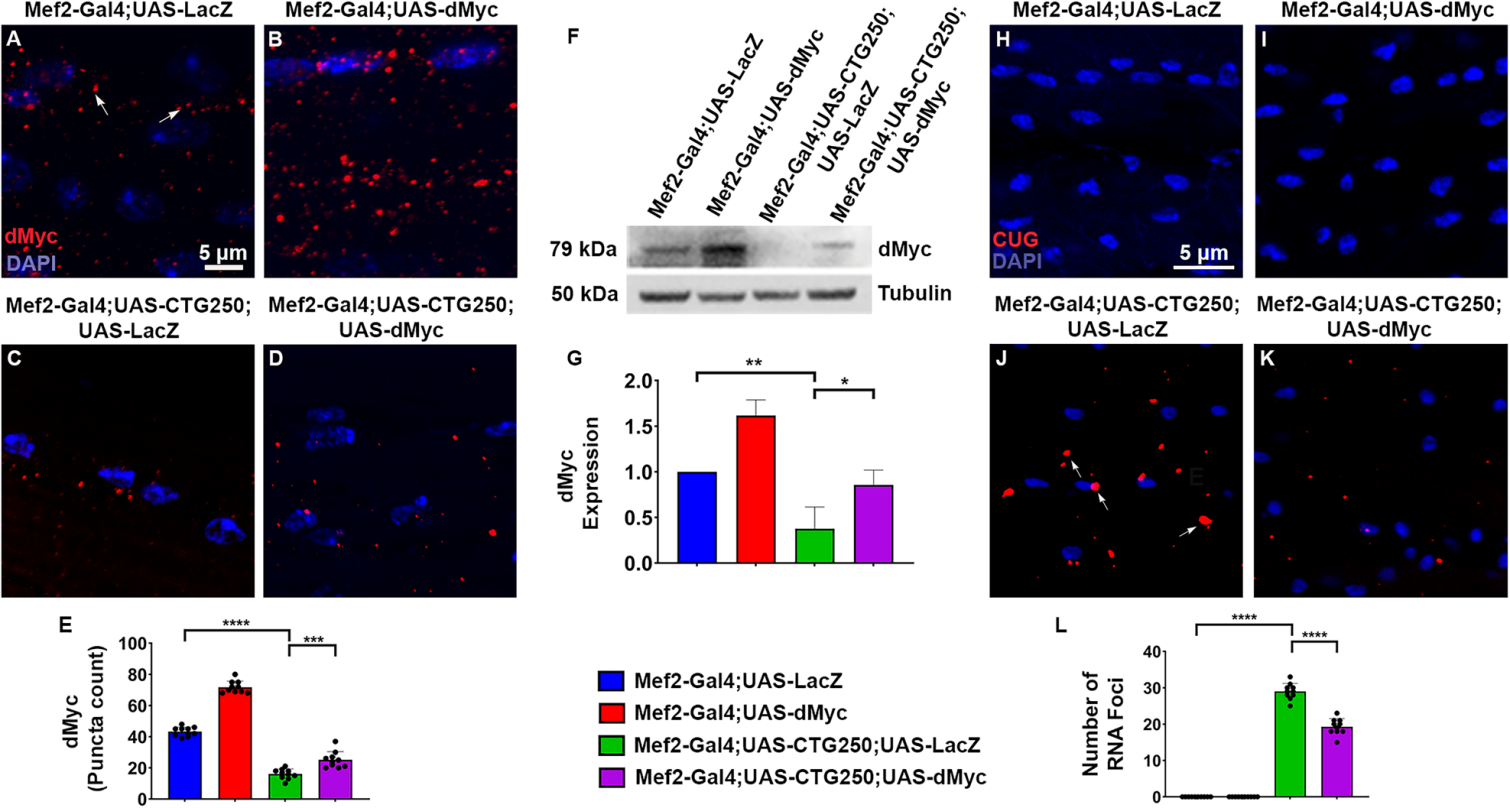
dMyc expression is impaired by CTG expansion, and its overexpression reduces RNA Foci. **A–D** Images of the immunofluorescent staining with anti-dMyc antibody in the hemithoraces of 3-day-old female flies and nuclei counterstained with DAPI (*n* = 10 flies/genotype). **A** Basal level of dMyc in the control flies. **B** Increased level of dMyc upon dMyc overexpression. **C** A significant decrease in dMyc level in the diseased flies. **D** dMyc level in the rescue flies. **E** Bar graph representing the dMyc expression level from the immunofluorescent staining results. **F** Western blot showing a significant reduction of dMyc protein expression in the diseased flies (*n* = 40 flies/genotype, *N* = 3). **G** Graph showing quantitative analysis of dMyc protein level in fly muscle tissue. **H–K** Images of the hemithoraces of the four genotypes under the control of the Mef2-Gal4. RNA foci were detected through FISH with a Cy5-labelled (CAG)_8_ RNA probe, and nuclei were counterstained with DAPI (*n* = 10 flies/genotype). **H**, **I** No RNA foci were observed in the control or dMyc overexpressing flies. **J** Several toxic RNA foci were observed in the disease flies. **K** There was a significant reduction in the number of RNA foci in the rescue flies. **L** Quantification graph showing reduction of RNA foci in the rescue flies. All bar graphs were presented as mean ± SD and analysed using one-way ANOVA with Tukey’s post-hoc analysis across genotypes. Statistical significance was indicated as follows: *P < 0.05, **P < 0.01, ***P < 0.001, ****P < 0.0001. Red: dMyc (**A–D**), RNA Foci (**H–K**), Blue: DAPI (**A**–**D**), (**H–K**). Scale bar (**A**–**D, H**–**K**): 5 µm.

Since the overexpression of dMyc improved the overall health of the DM1 model flies, we hypothesised that the dMyc levels could be altered in the CTG250 flies. Therefore, we investigated dMyc expression in CTG250 flies by immunohistochemistry using a specific dMyc antibody. Interestingly, our results showed a significant reduction in the dMyc signal in the thoracic muscles of disease flies compared with controls (Fig. 3A, C, E). The result was further validated by Western blotting, which showed a marked reduction in dMyc protein in disease flies (Fig. 3F, G). Overexpression of dMyc in the CTG250 flies resulted in increased dMyc protein level but at a lower expression than the control flies (Fig. 3A–G). However, improving its expression in the diseased flies could significantly suppress the CTG250 phenotype. This finding strongly suggests that dMyc expression may be impaired in DM1, and that modulating its expression may mitigate DM1 pathogenicity.

One of the pathological hallmarks of DM1 is the formation of toxic RNA foci composed of expanded poly (CUG) mRNA transcripts, which sequester RNA-binding proteins and disrupt normal splicing regulation [17–19]. Because dMyc could significantly improve the overall health of DM1 model flies and its expression is impaired, we wanted to assess its impact on RNA foci formation. We performed Fluorescence in situ Hybridisation (FISH) using a Cy5-labelled (CAG)_8_ RNA probe, which specifically detected expanded CUG-repeat-containing transcripts. No CUG RNA foci were detected in the control and dMyc-overexpressing flies (Fig. 3H, I, L). However, several CUG foci were observed in the thoracic muscle of CTG250 expressing flies (Fig. 3J). Interestingly, dMyc overexpression in CTG250 flies led to a marked reduction in RNA foci (Fig. 3K, L). We also checked for RNA foci in flies expressing CTG19 (non-pathogenic repeats); however, no RNA foci were observed (Fig. S5A, B). These findings suggest that dMyc may mitigate CTG250 toxicity by enhancing RNA processing or promoting the degradation of expanded transcripts, thereby alleviating a key molecular pathology associated with abnormally expanded CTG.

### dMyc reduces the aberrant autophagy level in the DM1 model flies

**Fig. 4:**
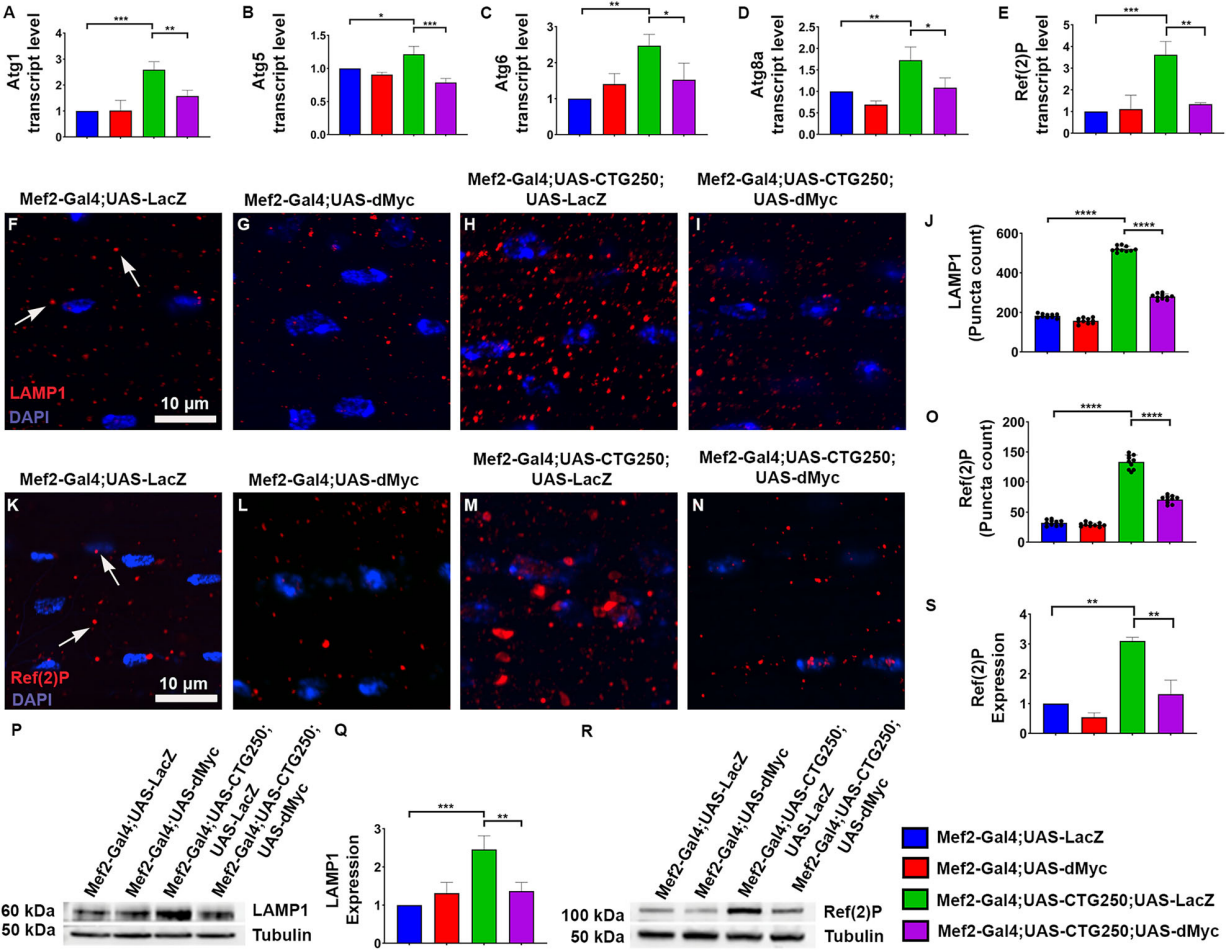
dMyc reduced the autophagy level in the CTG250 flies. **A–E** Quantitative PCR analysis of autophagy-related genes in thoracic tissues of the adult flies (*n* = 40 flies/genotype, *N* = 3). The expressions of Atg1, Atg5, Atg6, Atg8a, and Ref(2)P were found to be remarkably upregulated in the CTG250 flies. Overexpression of dMyc in the CTG250 background significantly reduced the expression closer to the control flies. **F–I** Immunofluorescence images of LAMP1 localisation in the hemithoraces of the flies (*n* = 10 flies/genotype). Nuclei were counterstained with DAPI. **F**, **G** Control and dMyc-overexpressing flies exhibited a baseline level of LAMP1 staining. **H** CTG250 flies displayed a marked increase in LAMP1 puncta, reflecting lysosomal accumulation consistent with impaired autophagy. **I** A significant reduction in LAMP1 staining was observed in the rescue flies, suggesting restoration of autophagy. **J** Quantification of LAMP1-positive puncta across the genotypes. **K–N** Images showing Ref(2)P signal (*n* = 10 flies/genotype). **K** Control and **L** dMyc-overexpressing flies displayed basal levels of Ref(2)P staining. **M** CTG250 flies showed elevated Ref(2)P levels. **N** Ref(2) P-positive puncta were significantly reduced in the rescue flies. **O** Quantification of Ref(2)P-positive puncta across all genotypes. **P** Western blot analysis of LAMP1 protein level across the genotypes showing marked elevation in disease flies (*n* = 40 flies/genotype, *N* = 3). This accumulation was substantially reduced in the rescue flies overexpressing dMyc. **Q** Quantification of LAMP1 immunoblots. **R** Furthermore, Western blot results showed a significant reduction of Ref(2)P in the rescue flies (*n* = 40 flies/genotype, *N* = 3). **S** Quantification of Ref(2)P immunoblots normalised to loading controls. All bar graphs were presented as mean ± SD and analysed using one-way ANOVA with Tukey’s post-hoc analysis across genotypes. Statistical significance was indicated as follows: *P < 0.05, **P < 0.01, ***P < 0.001, ****P < 0.0001. Red: LAMP1 (**F**–**I**), Ref(2)P (**K–N**), Blue: DAPI (**F**–**I**, **K**–**N**). Scale bar (**F**–**I, K**–**N**): 10 µm.

Studies have demonstrated that aberrant autophagy activation contributes to cellular dysfunction and muscle atrophy in DM1 and other neuromuscular disorders [20, 21]. To understand the underlying molecular mechanisms and to determine whether the rescue mediated by dMyc is through correcting aberrantly dysregulated autophagy, we assessed the expression levels of key autophagy genes Atg1, Atg5, Atg6, Atg8a, and Refractory to sigma, Ref(2)P, using quantitative real-time PCR. The results revealed a significant reduction of these transcripts in the rescue flies compared to the disease flies (Fig. 4A–E). We performed immunohistochemistry and Western blotting for Lysosomal-Associated Membrane Protein 1 (LAMP1) and Ref(2)P to further validate our findings. Both immunohistochemistry and Western blotting results showed a significant reduction in LAMP1 and Ref(2)P in the rescue flies compared with the disease flies, indicating normalisation of autophagic activity (Fig. 4H–J, M–O, P–S). The control and dMyc-expressing flies had basal levels of LAMP1 and Ref(2)P (Fig. 4F, G, J, K, L, O, P–S). We also used Atg8a-GFP to mark autophagosomes in the eye discs of flies expressing CTG270 repeats [22]. Diseased larval eye discs showed a marked accumulation of Atg8a-GFP positive puncta, which was significantly reduced in the rescue condition (Fig. S6A–E). Furthermore, we measured the autophagy flux using the GFP-mCherry-Atg8a reporter in the flies’ eye expressing CTG270 [22, 23]. In the disease condition, we observed an increase in both yellow (GFP+mCherry) autophagosomes and red-only autolysosomes, accompanied by a reduced GFP/mCherry ratio, indicating enhanced lysosomal fusion and elevated autophagic flux (Fig. S6L–N, R, S, T). In contrast, there was a decrease in both yellow and red puncta in the dMyc overexpressed flies and restored the GFP/mCherry ratio, demonstrating normalisation of the excessive autophagy flux rather than complete suppression of the pathway (Fig. S6Q–T). These findings suggest that dMyc may confer its protective effect by restoring autophagic balance, thereby mitigating abnormally expanded CTG-associated cellular pathology.

### dMyc restores the impaired cell death in the DM1 model flies

**Fig. 5:**
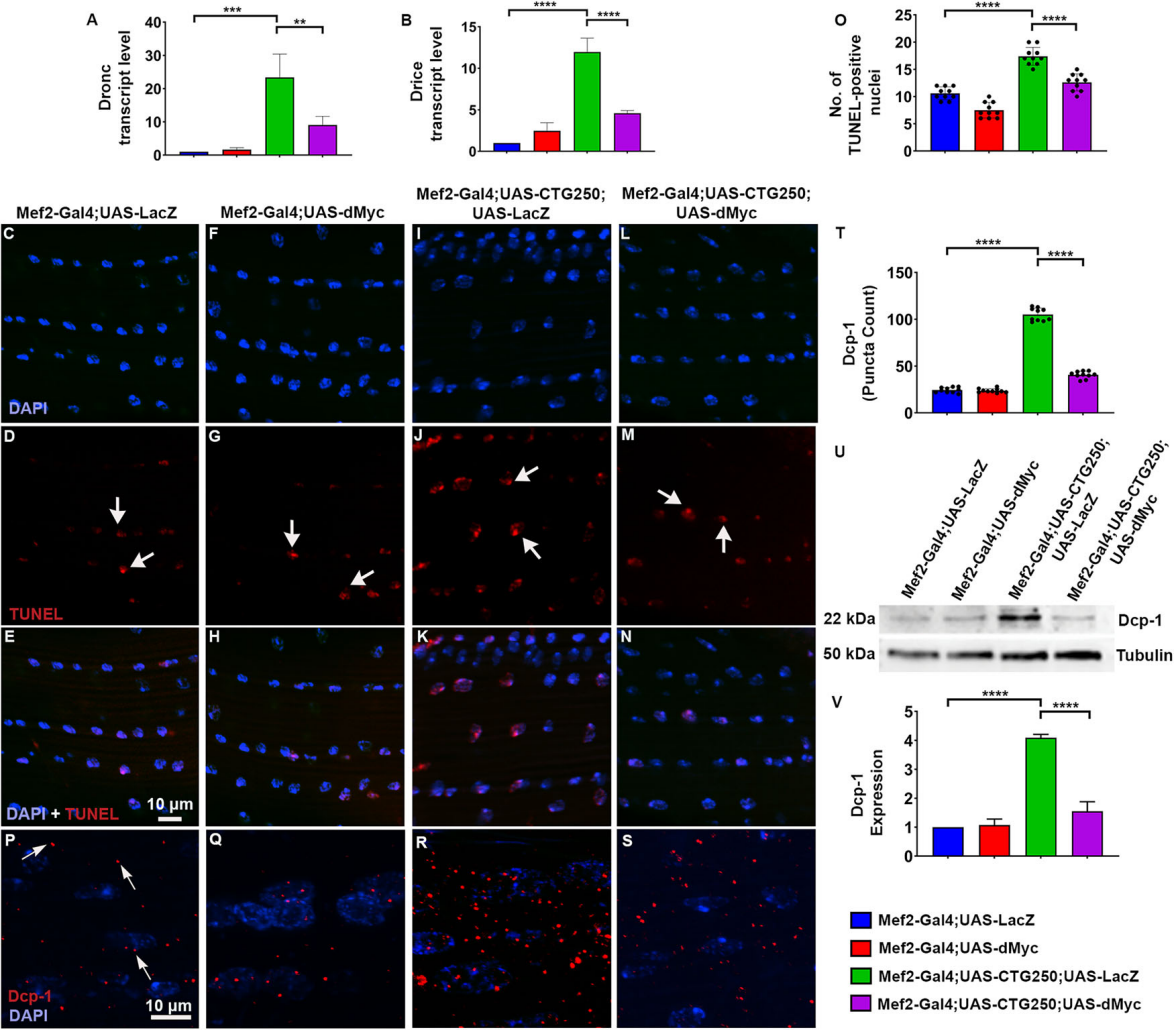
dMyc attenuates apoptosis in CTG250 flies. **A**, **B** Quantitative PCR analysis of apoptosis-related genes in thoracic tissues of adult flies (*n* = 40 flies/genotype, *N* = 3). The expression of Dronc and Drice was significantly upregulated in the CTG250 flies. Overexpression of dMyc in the CTG250 background significantly reduced the expression of these transcripts closer to the control flies. **C**–**N** Images showing TUNEL-positive nuclei (*n* = 10 flies/genotype, indicated by arrow). **C**–**E** Control and (**F**–**H**) dMyc-overexpressing flies showed basal levels of TUNEL-positive nuclei. **I**–**K** CTG250 flies showed elevated numbers of TUNEL-positive nuclei, indicating increased apoptosis. **L**–**N** However, TUNEL-positive nuclei were significantly reduced in the rescue flies. **O** Quantification of TUNEL-positive nuclei across the genotypes. **P**–**S** Immunofluorescence images of Dcp-1 signal in the hemithoraces of the flies (*n* = 10 flies/genotype). Nuclei were counterstained with DAPI. **P**, **Q** Control and dMyc overexpressing flies exhibited baseline levels of Dcp-1. **R** CTG250 flies displayed a marked increase in Dcp-1 signal. **S** A significant reduction in the Dcp-1 signal was observed in the rescue flies. **T** Quantification of Dcp-1 puncta count across the genotypes. **U** Western blot analysis showed a significant elevation of cleaved Dcp-1 in disease flies, which was remarkably reduced in disease flies with dMyc (*n* = 40 flies/genotype, *N* = 3). **V** Quantification graph of Dcp-1 immunoblots. All bar graphs were presented as mean ± SD and analysed using one-way ANOVA with Tukey’s post-hoc analysis across genotypes. Statistical significance was indicated as follows: **P < 0.01, ***P < 0.001, ****P < 0.0001. Red: TUNEL *P*ositive nuclei (**C**–**N**), Dcp-1 (**P**–**S**), Blue: DAPI (**C**–**N, P**–**S**). Scale bar (**C**–**N, P**–**S**): 10 µm.

Apoptosis has been reported to be a major contributing factor in DM1pathogenesis, where toxic RNA gain-of-function leads to cellular stress and activation of pro-apoptotic pathways [20]. In our study, as dMyc conferred a significant phenotypic improvement in the DM1 model flies, we hypothesised that dMyc may also exert its protective effects by attenuating cell death in disease flies. Therefore, we quantified transcript levels of key apoptotic genes, Death regulator Nedd2-like caspase (Dronc, homologue of human caspase-9) and Death-related ICE-like caspase (Drice, an effector caspase), to test this. The results showed significant upregulation of Dronc and Drice in CTG250 flies, which was markedly reduced upon dMyc overexpression (Fig. 5A, B). To further assess the reduction in DNA fragmentation and apoptosis in the rescue flies, we performed a Terminal deoxynucleotidyl transferase dUTP Nick-End Labelling (TUNEL) assay, which labels fragmented DNA in the nuclei of cells undergoing apoptosis. As hypothesised, a marked reduction in the number of TUNEL-positive nuclei was observed in the rescue relative to the disease flies (Fig. 5C–O). Additionally, we performed immunostaining of effector caspase Dcp-1. We observed that CTG250 flies exhibited a pronounced cleaved Dcp-1 signal, which was significantly reduced in the rescue flies (Fig. 5P–T). This finding was further validated by the Western blot, which confirmed a significant reduction in the elevated level of apoptosis in the rescue flies (Fig. 5U, V). Together with our findings on autophagy, these results suggest that dMyc ameliorates expanded CTG-associated cellular toxicity through dual suppression of both aberrant autophagy and apoptosis, thereby contributing to overall phenotypic rescue.

### Interaction of dMyc and autophagy-related genes modulates expanded CTG pathology

**Fig. 6:**
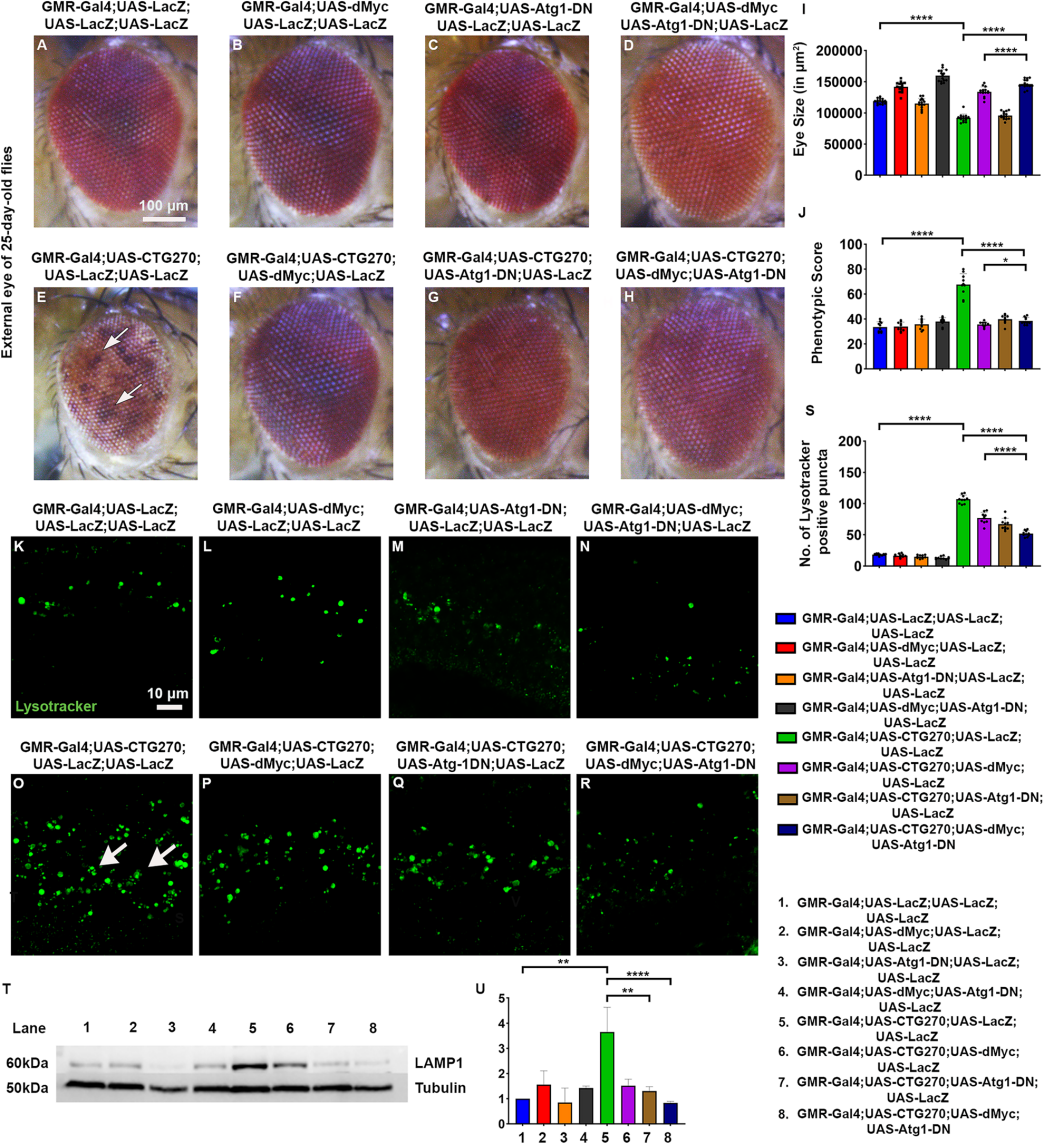
dMyc interacts with autophagy genes and modulates CTG-mediated phenotypes. **A**–**H** Bright-field eye images of 25-day-old female flies (*n* = 10 flies/genotype). **A** Eye image of the control fly. **B** Eye image of the dMyc overexpressed fly. **C** Eye of Atg1-DN fly. **D** Eye of dMyc and Atg1-DN overexpression fly. **E** Flies expressing CTG270 repeats exhibited a marked reduction in eye size and roughness, loss of pigmentation and formation of necrotic patches (indicated by an arrow). **F** dMyc overexpression improved eye size and pigmentation. **G** Expression of Atg1-DN in the disease background improved the CTG-mediated phenotype. **H** Co-expression of dMyc with Atg1-DN significantly improved the disease phenotype compared with either alone. **I** Bar graph showing the eye size. **J** Bar graph showing the phenotypic score of the eyes. **K**–**R** Confocal images of Lysotracker staining in the larval eye disc (*n* = 10 flies/genotype). **K**–**N** Basal level of autophagy observed in control, dMyc, Atg1-DN, and dMyc with Atg1-DN expressing flies. **O** CTG270 expressing flies showed a higher Lysotracker signal. **P**, **Q** Reduction of Lysotracker signal in the dMyc and Atg1-DN flies. **R** A significant reduction of Lysotracker signal in the flies’ eye co-expressing dMyc and Atg1-DN. **S** Bar graph showing quantification of Lysotracker signal. **T** Western blot showing reduced LAMP1 protein (*n* = 120 flies/genotype, *N* = 3). **U** Graph showing quantification of LAMP1 protein. All bar graphs were presented as mean ± SD and analysed using one-way ANOVA with Tukey’s post-hoc analysis across genotypes. Statistical significance was indicated as follows: *P < 0.05, **P < 0.01, ***P < 0.001, ****P < 0.0001. Green: Lysotracker (**K**–**R**). Scale bar (**A**–**H**): 100 µm, (**K**–**R**): 10 µm.

To determine whether dMyc interacts with autophagy genes to reduce aberrant autophagy in CTG-induced toxicity, we genetically combined dMyc overexpression with a dominant-negative allele of Atg1 (Atg1-DN) in the flies expressing CTG270 repeats. Adult eyes at 25 days revealed that co-expression of dMyc with Atg1-DN resulted in a marked improvement in eye size, morphology, and pigmentation compared with disease flies (Fig. 6E, H–J). Consistent with elevated autophagy in the disease condition, CTG270 flies exhibited increased LysoTracker staining and LAMP1 protein, which were significantly reduced upon co-expression of dMyc and Atg1-DN, indicating additive normalisation of autophagic activity (Fig. 6O, R–U). We further combined Atg1-RNAi and Atg8a-RNAi with dMyc overexpression, which similarly improved the eye phenotype in CTG270 flies (Fig. S7). Our results also show that modulation of the autophagy genes alone can mitigate the disease phenotype, which was further enhanced upon co-expression with dMyc (Figs. 6G–J, Q–U and S7I–N). Taken together, these results indicate that dMyc may play a crucial role in reducing elevated autophagy in the CTG-mediated pathogenesis.

### dMyc interacts with Diap1 to modulate CTG-mediated pathogenesis

**Fig. 7:**
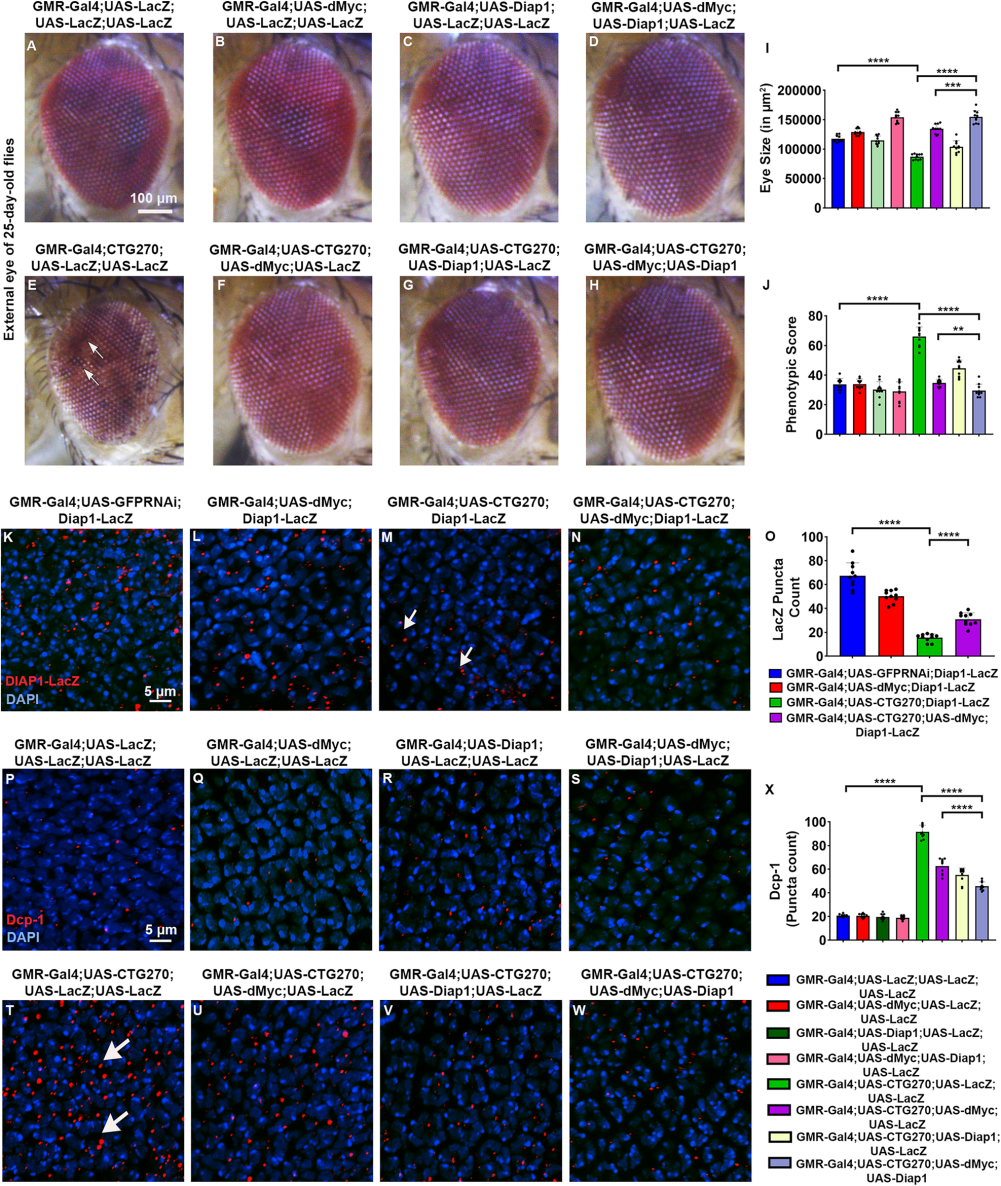
dMyc interacts with Diap1 and modulates CTG-expanded phenotypes. **A**–**H** Bright-field eye images of 25-day-old female flies (*n* = 10 flies/genotype). **A** Eye image of the control fly. **B** Eye image of the dMyc overexpressed fly. **C** Eye of Diap1 overexpressed fly. **D** Eye of dMyc and Diap1 overexpressed fly. **E** Flies expressing CTG270 repeats exhibited a marked reduction in eye size and roughness, formation of necrotic patches as well as loss of pigmentation (indicated by an arrow). **F** dMyc overexpression improved the disease eye phenotype. **G** Expression of Diap1 in the disease background improved the CTG-mediated phenotype. **H** Co-expression of dMyc with Diap1 could significantly improve the disease phenotype compared to either alone. **I** Bar graph showing the eye size. **J** Bar graph showing the phenotypic score of the eyes. **K**–**N** Confocal images showing Diap1 signal in the larval eye disc (*n* = 10 flies/genotype). **O** Bar graph showing a reduction of Diap1 puncta in the flies expressing CTG270, which was restored upon dMyc overexpression. **P**–**W** Confocal images of larval eye disc showing Dcp-1 puncta (*n* = 10 flies/genotype). There was a reduction of Dcp-1 puncta in the dMyc and Diap1 expressing larvae, which was further enhanced upon dMyc and Diap1 co-expression. **X** Bar graph showing quantification of Dcp-1 puncta. All bar graphs were presented as mean ± SD and analysed using one-way ANOVA with Tukey’s post-hoc analysis across genotypes. Statistical significance was indicated as follows: **P < 0.01, ***P < 0.001, ****P < 0.0001. Red: Diap1-LacZ (**K**–**N**), Dcp-1 (**P**–**W**), Blue: DAPI (**K**–**N, P**–**W**). Scale bar (**A**–**H**): 100 µm, (**K**–**N, P**–**W**): 5 µm.

Previous studies have shown that Myc regulates growth, development, and apoptosis (13, 14, 15). Our study reveals that dMyc overexpression reduces CTG-induced cell death. Therefore, to examine whether dMyc interacts with a cell death pathway gene and modulates CTG-mediated toxicity, we co-expressed dMyc with Death-associated protein inhibitor of apoptosis 1 (Diap1) in the eyes of disease flies. We observed a significant improvement in eye size, morphology, and pigmentation (Fig. 7E, G–J). Next, we used Diap1-LacZ reporter to determine the cell death [24]. We observed a pronounced reduction in Diap1 expression in the eye discs of the disease larvae expressing CTG270 (Fig. 7K–M, O). However, Diap1 expression was increased by dMyc overexpression (Fig. 7M–O). Expression of Diap1 reduced the Dcp-1 level in CTG270 flies (Fig. 7V, X). In addition, expression of both dMyc and Diap1 significantly reduced the Dcp-1 Level (Fig. 7W, X). These findings suggest that dMyc plays a functional role in reducing aberrant cell death in the DM1 disease model.

## Discussion

Myc is a master regulator of cell growth and metabolism, playing a critical role in the anabolic and catabolic processes of muscle tissue [25]. In our study, we identified dMyc as a potential modifier of DM1 pathogenesis. Various studies highlighted the role of Myc across multiple disease contexts, including cancer, neurodegenerative disorders, and muscular dystrophies [26–28]. In our study, we observed that Myc is downregulated in the flies expressing the abnormally expanded CTG repeats, and its overexpression mitigated the disease phenotype. A similar finding reported that dMyc ameliorated poly(Q) toxicity in a *Drosophila* model of Spinocerebellar Ataxia type 3 [29]. Therefore, the findings underscore the importance of exploring Myc’s therapeutic potential in DM1. In our study, we observed a significant improvement in motor activity and lifespan in CTG250 flies upon dMyc overexpression. This may be due to the restoration of actin and myosin protein arrangement in the diseased flies. The recovery of structural muscle proteins following dMyc overexpression highlights its critical role in maintaining muscle integrity and function. These findings align with previous studies suggesting low Myc levels may exacerbate muscle protein degradation in atrophic conditions [30–32]. Disruption of Myc signalling can impair muscle development or exacerbate atrophic phenotypes [33]. In a recent study, Myc overexpression in mouse skeletal muscle promotes cell growth by enhancing ribosomal RNA synthesis and the translational machinery through a mechanism independent of mTORC1 activation, suggesting that Myc can bypass traditional growth signalling pathways to stimulate muscle hypertrophy directly [30]. Transcriptomic meta-analyses of atrophying muscles have revealed that Myc-regulated gene networks may become dysregulated during muscle wasting [34]. As a multisystemic disorder, DM1 impairs several signalling pathways. A critical marker of energy metabolism, AMP-activated protein kinase signalling, is hampered by DM1. This signalling was restored through exercise or pharmacological interventions, thereby improving alternative splicing and the skeletal muscle phenotype in mice. [35, 36]. Another key pathway altered is insulin signalling, a key regulator of growth. A defect in the alternative splicing of the insulin receptor (IR) led to the predominant expression of IR-A, a non-muscle isoform with reduced signalling and a lower metabolic response to insulin, thereby causing insulin resistance in DM1 patients [37]. Within the Insulin/Akt/TOR signalling pathway, transcription factors such as FOXO, Myc, and Mnt coordinate muscle fibre growth. Activation of the insulin pathway, for example, by overexpressing the insulin receptor, leads to an upregulation of Myc, which stimulates nucleolar biogenesis and facilitates muscle growth [38–40]. Experimental downregulation of Myc via RNAi impairs muscle growth, highlighting its essential role in myogenesis [38]. Importantly, activation of the Insulin/Akt pathway has been shown to prevent denervation-induced muscle loss, suggesting a potential therapeutic role for Myc, which is downstream of this pathway [40, 41]. Our study showed a significant reduction in RNA foci formation in dMyc-overexpressing CTG-expressing flies. Previous studies have shown that modifying aberrant splicing or enhancing RNA decay pathways can alleviate DM1 phenotypes [42]. Since RNA foci formation is central to DM1 pathogenesis, their reduction suggests that dMyc may improve RNA processing or accelerate the degradation of toxic RNA transcripts. Factors contributing to muscle atrophy in DM1 are the abnormal increase in autophagy and apoptosis [20, 43]. Skeletal muscle biopsies from DM1 patients have also shown the downregulation of autophagy and apoptosis repressor genes. Repression of autophagy and apoptosis pathways reduced muscle atrophy in *Drosophila* and human DM1 samples [20]. A positive correlation has been found between the length of CTG expansion and the activation level of apoptosis and autophagy in primary cultures of human DM1 skeletal muscle [44]. Our results show a significant reduction in autophagy in the rescue flies, highlighting Myc’s role as an autophagy modulator. Our finding correlates with a previous study that shows Myc suppresses autophagy by blocking the function of Transcription Factor EB, a key regulator of autophagy and lysosome biogenesis [45]. Under physiological conditions, Myc is necessary for maintaining basal autophagy, as Myc inhibition disrupts autophagosome formation, indicating that a certain level of Myc activity is essential for autophagy homoeostasis [46]. These findings suggest a dual role for Myc: while its complete loss impairs necessary autophagy, its overexpression may suppress pathological or excessive autophagy. Our results also show a marked reduction of the aberrant apoptosis in the rescue flies. Myc has an established role in regulating apoptosis by modulating the balance between pro-survival and pro-apoptotic signals in the B-cell lymphoma-2 (BCL-2) pathway [47]. In this context, Myc may exert a protective role by attenuating aberrant cell death associated with CTG pathogenesis in the DM1 model. Our overall findings suggest a potential role of dMyc in DM1 pathogenesis. Although Myc is an oncogene, its tightly regulated expression may offer a strategy to combat degenerative changes without triggering tumorigenesis. The study highlights the future direction in understanding DM1 and presents a promising approach for developing targeted interventions.

## Materials and methods

### *Drosophila* stocks and genetics

All the fly stocks were maintained on standard cornmeal-sucrose-yeast-agar medium under a 12:12 h light-dark cycle at 25 ± 0.5 °C. The following fly lines were procured from the Bloomington Drosophila Stock Center (BDSC, Indiana University, Bloomington, Indiana, USA): GMR-Gal4 (1104), Mef2-Gal4 (27390), UAS-DsRed-CTG250 (79580), UAS-DsRed-CTG270 (79581), UAS-DsRed-CTG19 (79576), UAS-dMyc (9674), UAS-Atg1-DN (60735), UAS-Atg1-RNAi (26731), UAS-Atg8a-RNAi (80428), UAS-Diap1 (6657), Diap1-LacZ (12093), UAS-Atg8a-GFP (51656), and UAS-GFP-mCherry-Atg8a (37749). UAS-LacZ was used as a transgenic control. We utilised GMR-Gal4 and Mef2-Gal4 to investigate the phenotype in the eyes and muscles of the DM1 fly models.

### Imaging and quantification of adult eye phenotype

High-resolution bright-field images of adult *Drosophila* eyes were obtained using a Leica M165 C stereo zoom microscope equipped with a 1X Plan objective at 5.0X magnification. For imaging, 25-day-old female flies from all four genotypes were taken. Flies were anaesthetised and mounted on glass slides by applying transparent nail polish. Each fly was carefully positioned such that one compound eye faced upward to optimise image acquisition. Images were captured using the Leica Application Suite X (LAS X) software with standardised settings: 57% brightness, gamma (γ) at 1.23, and saturation at 1.15. Post-acquisition, eye size was quantified using ImageJ software (National Institutes of Health, Bethesda, MD, USA) as previously described [48]. The perimeter of each eye was manually outlined using the segmented freehand line tool, and the enclosed area was measured using the “Measure Area” function to assess differences in eye size across genotypes. The phenotypic score of the eyes was quantified using the Flynotyper plugin [16].

### Larval crawling assay

Third-instar female larvae of each genotype were washed with PBS to remove food traces and then placed on blotting paper soaked in PBS. Three larvae were then acclimatised on an agar plate for 1 min before recording a 1 min video at 30 frames per second, 1080 pixels, using a recording device with a 13-megapixel camera (lens aperture f/2.2, wide-angle lens, 1.12 µm Camera pixel, Phase Detection Auto Focus). This experiment was repeated 15 times for each genotype. The video was converted to grayscale, cropped to focus on the plate area, and the image stack was inverted to enhance the visualisation of the larvae. A substack of 1000 frames was created, and the scale was set to pixel/mm based on the previously known plate diameter. Background subtraction and image thresholding were performed to highlight the larvae against the background. The WrMTrck plugin was then used for analysis, with parameters set to track the larvae’s movement, including size, velocity, area change, and track length, as previously done [49, 50]. This analysis provided data on the distance travelled by the larvae and their speed within the specified time frame, with a tracing of path length for comparison.

### Negative geotaxis assay

The climbing ability of adult flies was assessed using a negative geotaxis assay, which leveraged Drosophila’s innate tendency to move against gravity [51]. The assay was performed on 3-day-old female flies to evaluate motor function across different genotypes. Groups of ten flies were gently transferred into standard vertical climbing assay vials marked at a height of 8 cm from the base. Following a 5 min acclimatisation period, flies were tapped down to the bottom of the vial, and the number of flies that successfully climbed above the 8 cm mark within 10 s was recorded. Each group underwent 6 trials, with a 1 min rest period between trials to prevent fatigue. For each genotype, approximately 120 flies were tested across multiple replicates. The percentage of flies reaching the 8 cm mark was calculated for each trial and averaged across replicates. Graphical representation and statistical testing were performed using GraphPad Prism version 10.5.0.

### Longevity assay

A survival assay was conducted on newly enclosed female *Drosophila*. An approximate of 150 flies were stochastically and equally divided into ten vials, each with ten flies. The flies were transferred to fresh vials every other day, with the number of dead flies recorded each time, until all the flies were dead. The study was concluded when all the flies were dead, and the survival curve was plotted versus time. The mean lifespan of flies was calculated using a Log-Rank statistical test and plotted (Kaplan–Meier plot) using the online application for survival analysis (OASIS 2) software [52].

### Immunostaining

In general, 3-day-old female flies were used for muscle immunostaining in all the experiments. The thoraces of the flies were separated from the abdomen, head, and legs using Dumont forceps (Cat. No. 11254-20, FST, Switzerland) in phosphate-buffered saline (PBS) and fixed for 2 h in 1x PBS with 4% paraformaldehyde (PFA). Thoraces were washed with PBST (0.1% Triton X-100) three times for 10 min each. After this, the thoraces were neatly dissected in half using a razor blade (Gillette Wilkinson Sword, platinum-coated) after being snap-frozen in Liquid Nitrogen for 10 s. Tissues were then blocked with a blocking buffer (PBS containing 0.1% BSA) for 2 h, followed by overnight incubation with the primary antibody at 4 °C. The following primary antibodies were used: mouse anti-Myosin Heavy Chain (1:200, 3E8-3D3, Developmental Studies Hybridoma Bank, Iowa, USA), Alexa Fluor 488 Phalloidin (1:100, A-12379, ThermoFisher Scientific, USA), mouse anti-dMyc (1:20, P4C4-B10, Developmental Studies Hybridoma Bank, Iowa, USA), rabbit anti-LAMP1 (1:200, ab30687, Abcam, UK), rabbit anti-Ref(2)P (1:200, ab178440, Abcam, UK), anti-LacZ (1:200, Developmental Studies Hybridoma Bank, Iowa, USA) and anti-Dcp-1 antibody (1:100, 9578, Cell Signalling Technology, USA). Samples were again washed with PBST 3 times for 10 min each, then incubated with fluorophore-conjugated secondary antibodies for 2 h in the dark at room temperature. Fluorophore-conjugated secondary antibodies used were Alexa 647 goat anti-mouse (1:200, A-32728, ThermoFisher Scientific, USA) and Alexa 647 goat anti-rabbit (1:200, A-21245, ThermoFisher Scientific, USA). The tissues were washed in PBST and counterstained with DAPI (5 μg/ml, D3571, Invitrogen, USA). They were then mounted using Vectashield mounting media (Vector Laboratories, USA) and imaged using a Nikon Confocal Microscope (Model AXR, Tokyo, Japan) with 20×, 40×, 60× oil-immersion, and 100× oil-immersion objectives. Image acquisition, processing, and analysis were performed using NIS-Elements C and ImageJ.

### Atg8aGFP and GFP-mCherry-Atg8a puncta measurement

Third-instar female larval eye discs were dissected in cold phosphate-buffered saline (PBS) and fixed in 4% paraformaldehyde (PFA) for 2 min, followed by three washes with PBS. The tissues were immediately mounted and imaged on the same day using a confocal microscope (AXR, Nikon, Tokyo, Japan). Image acquisition, processing, and analysis were performed using NIS-Elements C software and ImageJ. Images were acquired at a resolution of 1024 × 1024 pixels for quantitative analysis of GFP and mCherry puncta. For figure presentation, representative regions were cropped to 512 × 512 pixels to enhance puncta visibility. Autophagic structures were quantified by counting GFP-positive, mCherry-positive, and GFP+mCherry (yellow) puncta. The GFP/mCherry puncta ratio was calculated to evaluate autophagy flux [22].

### Lysotracker staining

Third instar female larval eye discs were dissected in cold PBS, fixed with 4% PFA for 2 min, and washed three times with PBS. The samples were stained with the Lysotracker Green (1:100 dilution, Catalogue no. L7526, ThermoFisher Scientific, USA) for 2 min. After washing, the eye discs were mounted and examined via confocal microscopy (Model AXR, Tokyo, Japan) on the same day. Image acquisition, processing, and analysis were conducted using NIS-Elements C and ImageJ.

### RNA-fluorescent in-situ hybridisation (RNA-FISH)

Thoraces of 3-day-old female flies were dissected into hemithoraces in PBS, rinsed with methanol, and then washed with methanol: PBST in a 1:1 ratio for 2 min. Samples were washed twice with PBST for 2 min each, then fixed for 20 min in PBS containing 4% PFA, and finally washed again with PBST three times for 2 min each. Permeabilization was performed using 1 μg/ml Proteinase K in PBS for 10 min at room temperature. Afterward, the samples were immediately transferred to ice and incubated for an additional 60 min. This was followed by two 2 min washes in 2 mg/ml Glycine in PBST to neutralise the Proteinase K activity. Samples were fixed again in 4% PFA in PBS for 20 min with gentle shaking, followed by five washes with PBST (each for 2 min). Tissues were then rinsed with a 1:1 mixture of PBST and hybridization solution (HS) (50% formamide, 20× SSC, 0.1% Tween-20, 100 g/ml Deoxyribonucleic acid from herring sperm). Pure HS (100%) was then added to the samples, which were incubated at room temperature. Meanwhile, the HS was preheated to 80 °C for 5 min and then immediately cooled on ice for 5 min to prepare the pre-hybridisation mix. Two hundred microlitres of pre-hybridization solution was added to each genotype, and the mixture was incubated at 56 °C for 2 h. For probe hybridisation, 200 ng of Cy5-labelled (CAG)_8_ RNA probe (Integrated DNA Technology, USA) was diluted in 200 μl of HS, denatured at 80°C for 3 min, and immediately chilled on ice for 5 min. This probe solution was then added to the tissue samples and incubated overnight at 56 °C.

The following day, tissues were rinsed briefly (1–2 min) in 100% HS, followed by sequential 15 min washes with the following solutions: 100% HS, 3:1 HS:PBST, 1:1 HS:PBST, and 1:3 HS:PBST. This was followed by four final washes with PBST (each lasting 5 min). Samples were maintained at room temperature and mounted using Vectashield mounting medium containing DAPI (Vector Laboratories, USA). Imaging was performed using a Nikon AXR confocal microscope (Model AXR, Tokyo, Japan) using a 40× objective, and up to 4× optical zoom. Image acquisition and processing were conducted using NIS-Elements C software.

### Terminal deoxynucleotidyl transferase dUTP nick-end labelling (TUNEL) assay

Apoptotic cells in 3-day-old female *Drosophila* muscle tissue were detected using the FITC-conjugated TUNEL label mix (Roche Catalogue no. 11767291910, Indianapolis, USA) and the TUNEL enzyme (Roche Cat no. 12156792910; Indianapolis, USA) according to the manufacturer’s protocol. Thoraces were dissected into hemithoraces from adult female flies and fixed in 4% paraformaldehyde (PFA) in PBS for 15 min at RT. Following fixation, hemithoraces were washed twice with PBS for 5 min each. The tissues were immersed in 1 ml of 0.1 M Citrate buffer, pH 6, for 5 min. Then, the tissues were microwave irradiated for 1 min, followed by immediate cooling by adding double-distilled water at room temperature. The tissues were washed with PBS and then immersed in 0.1 M Tris-HCl, pH 7.5, containing 3% BSA and 20% FBS for 30 min at room temperature. The tissues were rinsed twice with PBS. TUNEL labelling was performed by incubating samples with 45 µl of FITC-conjugated TUNEL label mix and 5 µl of TUNEL enzyme at 37 °C in the dark for 48 h, followed by a PBS wash. Finally, samples were mounted in Vectashield mounting medium with DAPI (Vector Laboratories, USA) to stain nuclei. Fluorescent images were acquired using a Nikon AXR confocal microscope (Model AXR, Tokyo, Japan). Image acquisition and processing were performed using NIS-Elements C imaging software.

### RNA isolation and real-time RT-PCR

Total RNA extraction was performed from 3-day-old female *Drosophila* thoraces (*n* = 40) using TRIzol reagent (Cat. No. T9424, Sigma-Aldrich, USA) according to the manufacturer’s protocol. The cDNA was synthesised from 1 μg of total RNA using the iScript cDNA Synthesis Kit (cat no. 1708891, Bio-Rad, USA). Real-time PCR was performed using TB Green Premix Ex Taq II (Cat no. RR820A, Takara, Japan) in the CFX-96 Bio-Rad PCR machine (Bio-Rad, USA). The amplification reactions were run in triplicate. The ribosomal protein 49 (rp49) was used as the loading control. Statistical analysis was performed using 2^−ΔΔCq^ and presented as mean ± standard deviation. The corresponding primer sequences used were:

*rp49 (F)*: 5’-ATGCCCAACATCGGTTAC-3’

*rp49 (R)*: 5’-CAATCTCCTTGCGCTTCT-3’

*Atg1 (F)*: 5’-CGTCTACAAAGGACGTCATCGCAAGAAAC-3’

*Atg1 (R)*: 5’-CGCCAAGTCGCCGCCATTGCAATACTC-3’

*Atg5 (F):*5’-CCTGCGAATCTATACAGACGATGAC-3’

*Atg5 (R):*5’-AGCTCAGATGCTCGGACATCCATTG-3’

*Atg6 (F):*5’-GGAGTTATCTTTGCCCATC-3’

*Atg6 (R):*5’-TAGAGTCCGTAAGCCTGT-3’

*Atg8a* (F): 5’-TCGCAAATATCCAGACCGTGTGCCCGTC-3’

*Atg8a*(R): 5’-GCCGATGTTGGTGGAATGACGTTGTTCAC-3’

*Ref(*2*)P (F):*5’-CGTAAGGACCTTCTGGATCG-3’

*Ref(*2*)P (R):*5’-GTGCATATTGCTCTCGCACT-3’

*Dronc (F):*5’-TGGTGAACAAGCCGAAGG-3’

*Dronc (R):*5’-GCATAGCAGACCAGAGTGTC-3’

*drICE (F):*5’-CTACGCCAAGGACACACAG-3’

*drICE (R):*5’-GGAGTCGCCATCGGTTTC-3’

### *Drosophila* lysate preparation and Western blotting

Thoraces of 3-day-old and eyes of 15-day-old female flies were homogenised in ice-cold RIPA buffer [0.5% Triton X-100, 150 mM NaCl, 50 mM Tris, 0.4% Na-Azide, 2% SDS, 10% Na-deoxycholate, phosphatase inhibitor (A32957, ThermoFisher Scientific, USA) lx, protease inhibitor (1,862,209, ThermoFisher Scientific, USA) 5 µl/ml, pH 7.2]. The homogenate was centrifuged at 14,000 rpm for 20 min at 4 °C, and the resulting supernatant solution was isolated. Subsequently, 20 µg of protein from each sample was run through SDS-PAGE. The protein samples were transferred to a 0.2- or 0.45-μm pore-sized nitrocellulose membrane (Cat. nos. 1620112 and 1620115, Bio-Rad, USA). Followed by a 1 h blocking incubation with 5% skimmed milk with PBST, the immunoblots were initially probed with anti-dMyc antibody (1:60, P4B4C10, Developmental Studies Hybridoma Bank, Iowa, USA), anti-Dcp-1 antibody (1:500, 9578, Cell Signalling Technology, USA), anti-β-actin antibody (1:1000, JLA20, Developmental Studies Hybridoma Bank, Iowa, USA), anti-Myosin antibody (1:1000, 3E83D3, Developmental Studies Hybridoma Bank, Iowa, USA), anti-Ref(2)P (1:1000, ab178440, Abcam, UK), rabbit anti-LAMP1 (1:1000, ab30687, Abcam, UK), and anti-β-Tubulin antibody (1:1000, E7, Developmental Studies Hybridoma Bank, Iowa, USA). The blots were incubated with either anti-mouse HRP (1:5000, 31430, ThermoFisher Scientific, USA) or anti-rabbit HRP (1:5000, 31460, ThermoFisher Scientific, USA) secondary antibodies. The blot was developed using SuperSignal West Pico PLUS Chemiluminescent Substrate (ThermoFisher Scientific, USA). Quantification of immunoblot signals was done using ImageJ software (NIH).

### Statistical analysis

All statistical analyses and graph generation were performed using GraphPad Prism (version 10.5.0). Depending on the experimental design, data were analysed using one-way analysis of variance (ANOVA), followed by Tukey’s multiple comparison post hoc test to identify statistically significant differences among groups. Results were expressed as mean ± SD. A *p*-value of less than 0.05 was considered statistically significant. The level of significance was represented in the graphs as follows: ns = non-significant, *P < 0.05, **P < 0.01, ***P < 0.001, ****P < 0.0001.

Supplementary information is available at Cell Death Discovery’s website.

## Supplementary information

Video 1

Video 2

Video 3

Video 4

Video 5

Supplemental Material

Original Western Blots

## Data Availability

The datasets generated and analysed during the current study are available from the corresponding author on reasonable request.
