# Supplementary material for: dMyc suppresses CTG-induced cytotoxicity in the Drosophila model of DM1 by reducing autophagy and cell death
Source: Cell Death Discov. 2026 Apr 20;12:261. doi: 10.1038/s41420-026-03123-w (PMC13223253; doi:10.1038/s41420-026-03123-w)
Supplement: Supplementary file 6 — Supplemental Material [file 41420_2026_3123_MOESM6_ESM.docx]

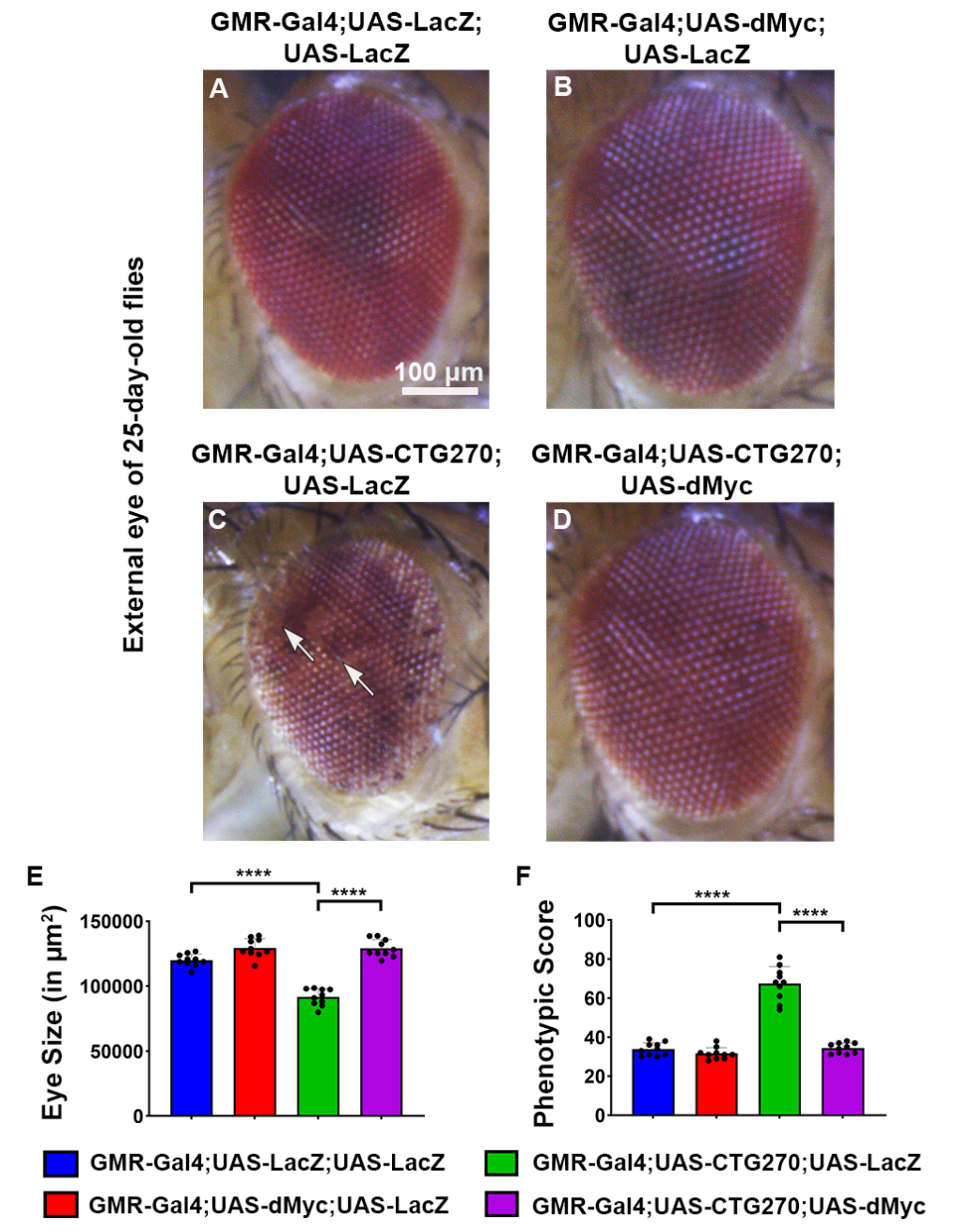


**Fig. S1.** dMyc improved the external eye phenotype of the CTG270 flies. (**A-D**) Bright-field images of the 25-day-old female adult fly eye (n=10 flies/genotype). (**A**) Eye image of the control fly. (**B**) Eye image of the dMyc overexpressed fly. (**C**) Flies expressing CTG270 repeats exhibited reduced eye size and loss of pigmentation with the formation of the necrotic patches (indicated by arrow). The eyes showed necrotic patches (indicated by the arrow). (**D**) dMyc overexpression led to significant improvements in eye size and pigmentation in disease flies. (**E**) Bar graph comparing eye size between different genotypes. (**F**) Bar graph comparing the Phenotypic score between different genotypes using Flynotyper (n=10 flies/genotype). All bar graphs were presented as mean ± SD and analyzed using one-way ANOVA with Tukey’s post-hoc analysis across genotypes. Statistical significance was indicated as follows: *****P* < 0.0001. Scale bar (**A-D**): 100 µm.


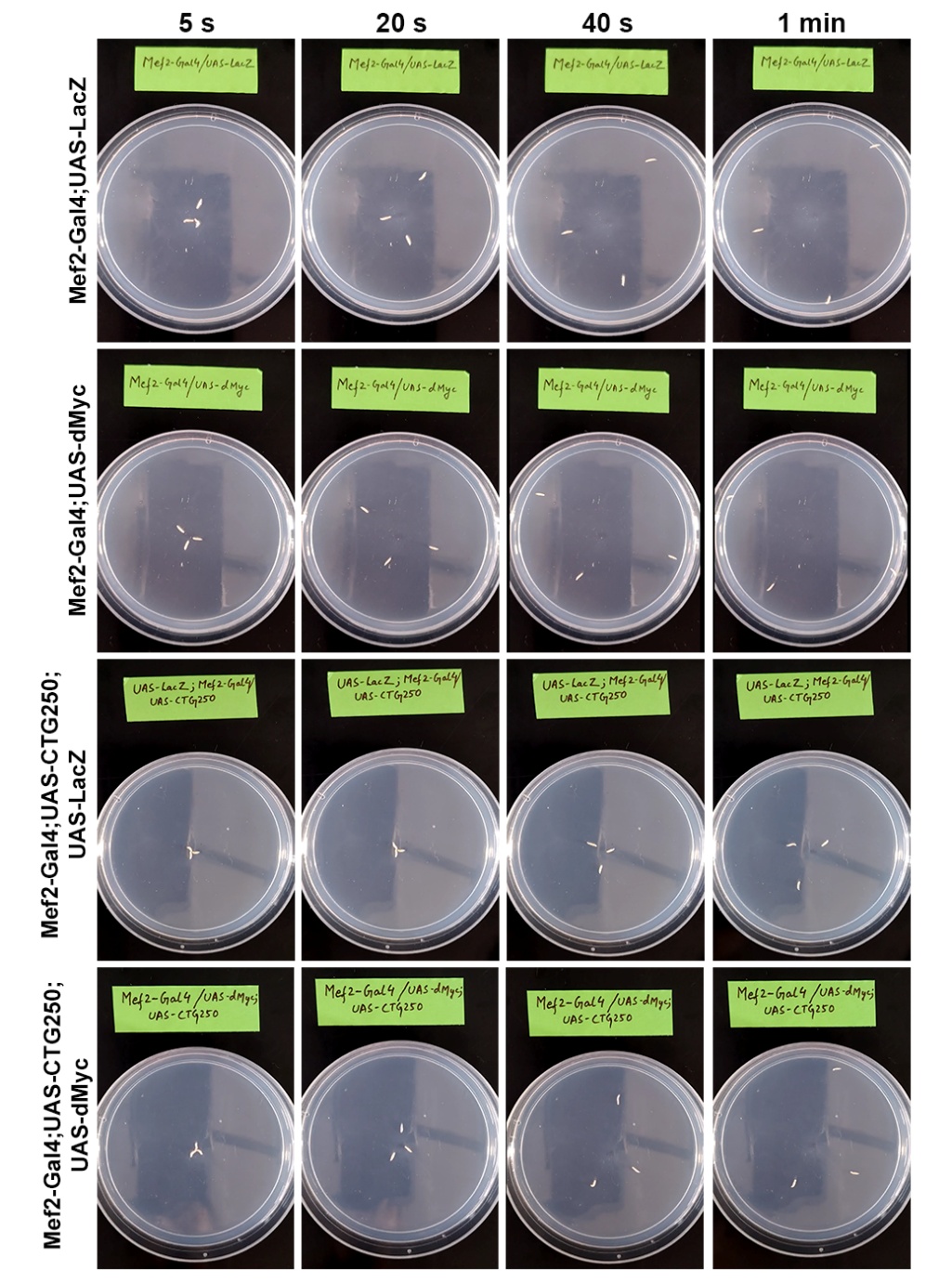


**Fig. S2.** dMyc improved the crawling ability of the disease larvae. Representative images of the larval crawling at 5 s, 20 s, 40 s, and 1 min of the video across the genotypes.


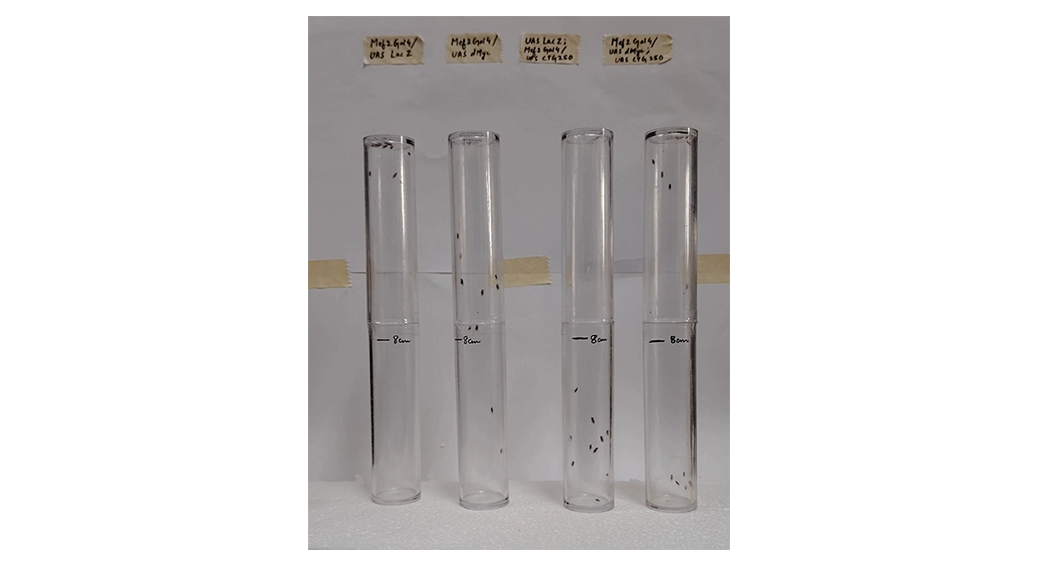


**Fig. S3.** dMyc improved the climbing ability of the disease flies. Overexpression of CTG250 caused a dramatic reduction in climbing ability, which is improved by overexpression of dMyc. Representative image of the climbing at 20 s of the videos.


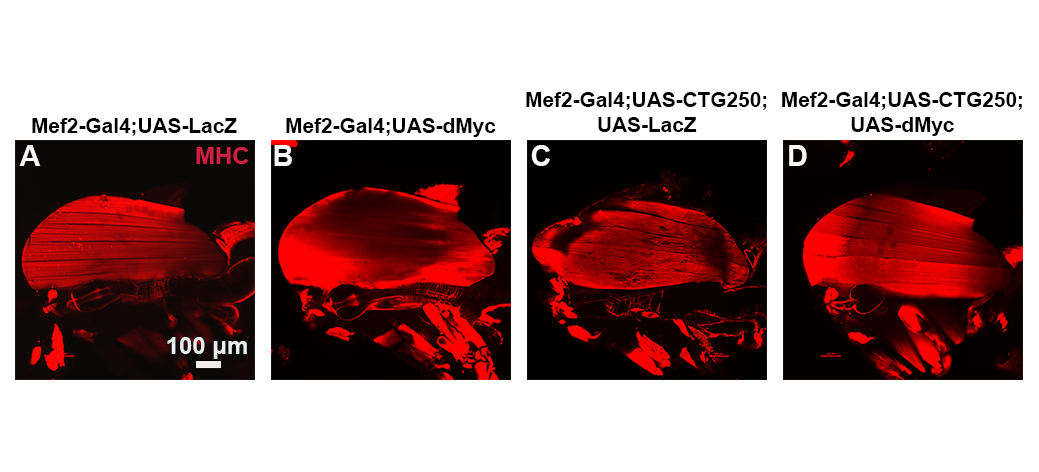


**Fig. S4**. Overexpression of dMyc improved the muscle architecture of the *Drosophila* Dorso-lateral flight muscles (DLMs). (**A-D**) Hemi-thoraces of 3-day-old flies with different genotypes under the control of the Mef2-Gal4, exposing the DLMs of the IFMs, and imaged using confocal microscopy (n=10 flies/genotype). Myosin filaments were stained with MHC and visualized in red to assess muscle structure. (**A-B**) Proper arrangement of the Myosin filaments with clear and distinct DLMs, in the control and dMyc overexpressed flies. (**C**) Disease flies exhibited disorganized myosin bands, indicative of disrupted muscle architecture. (**D**) dMyc overexpression improved the organisation of myosin bands in the disease flies. Red: MHC. Scale bar: 100 µm.


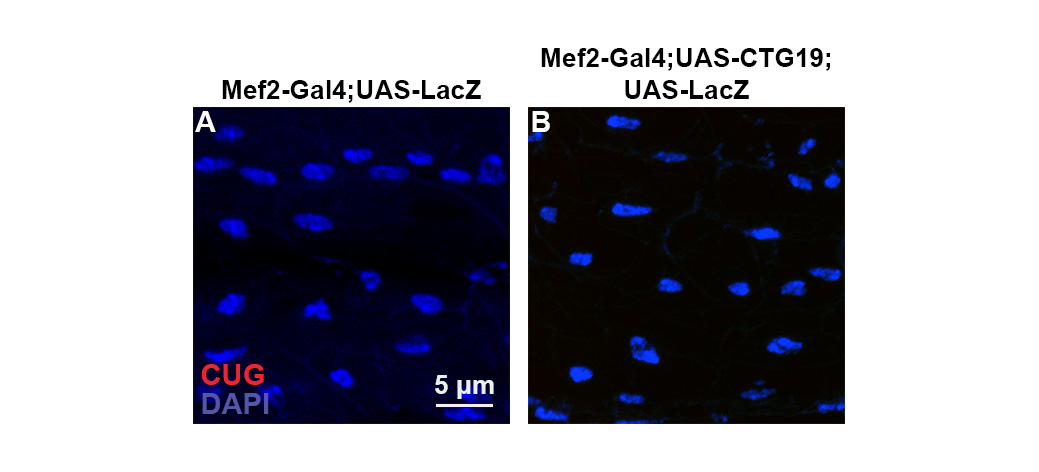


**Fig. S5**. No RNA foci were detected in the thoracic muscle of the flies expressing CTG19. (**A**) Control flies without any foci. (**B**) No RNA foci were detected in the muscle sections of CTG19 flies. Red: RNA Foci, Blue: DAPI. Scale bar: 5 µm.


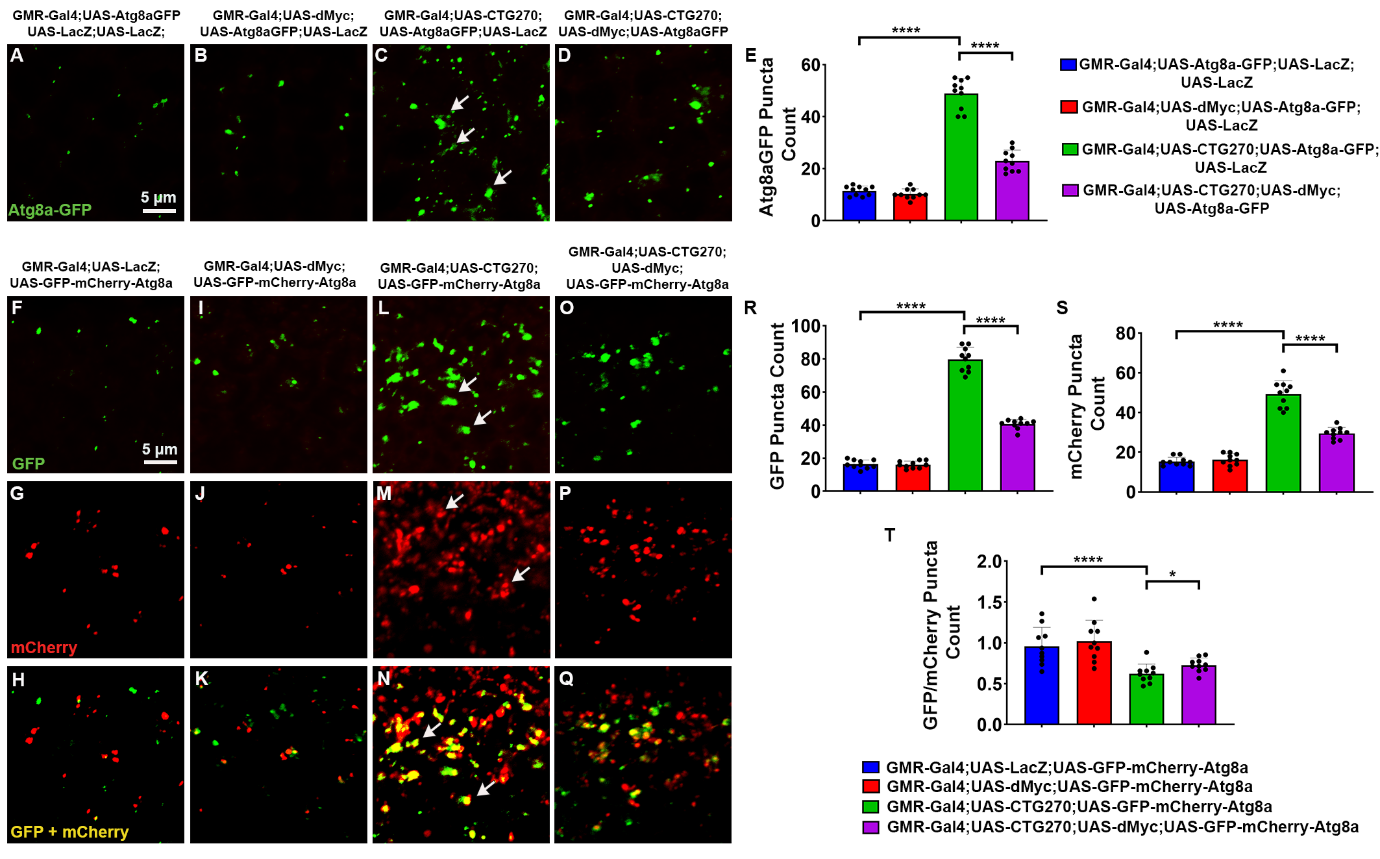


**Fig. S6.** dMyc reduces aberrant autophagy in the eye of the flies' expressing CTG270. (**A-D**) Representative images showing Atg8a-GFP puncta. (**A**) Control flies. (**B**) dMyc overexpressed flies. (**C**) Disease flies show increased accumulation of Atg8a-GFP puncta, indicating increased formation of autophagosomes. (**D**) Decrease in Atg8a-GFP puncta in the rescue flies. (**E**) Graph showing Atg8a-GFP puncta count. (**F-Q**) Representative confocal images showing GFP-mCherry-Atg8a puncta in the larval eye disc. (**F-H**) Basal level of autophagy in the control fly. (**I-K**) Basal autophagy level in dMyc overexpressed flies. (**L-N**) The disease flies show an increase in both red autolysosomes and yellow (GFP + mCherry) autophagosomes, and a decrease in the GFP/mCherry ratio, indicating excessive lysosomal fusion and increased autophagy flux. (**O-Q**) dMyc overexpression in the disease flies resulted in a reduction of both the yellow and red puncta and the GFP/mCherry ratio, indicating the reduction in autophagy flux. (**R**) Graph showing GFP puncta count. (**S**) Graph showing mCherry puncta count. (**T**) Graph showing GFP/mCherry puncta count. All bar graphs were presented as mean ± SD and analyzed using one-way ANOVA with Tukey’s post-hoc analysis across genotypes. Statistical significance was indicated as follows: **P* < 0.05, *****P* < 0.0001. Green: UAS-Atg8a-GFP (**A-D**), UAS-GFP-mCherry-Atg8a (**F, I, L, O, H, K, N, Q**). Red: UAS-GFP-mCherry-Atg8a (**G, J, M, P, H, K, N, Q**). Yellow: GFP + mCherry (**H, K, N, Q**). Scale bar (**A-D, F-Q**): 5 µm.


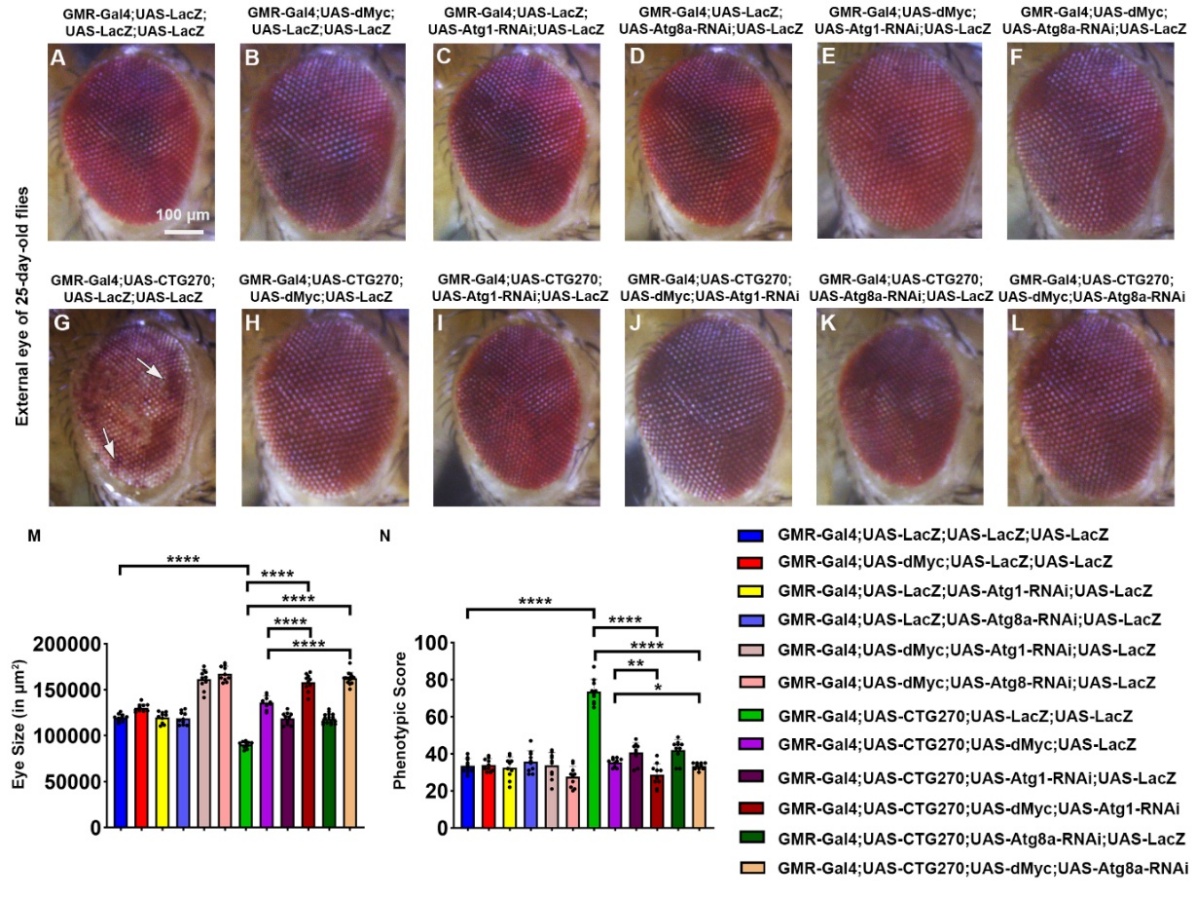


**Fig. S7.** Knockdown of Atg genes ameliorates the CTG270 eye phenotype. (**A-L**) Bright-field images of the 25-day-old adult fly eye (n=10 flies/genotype). (**A**) Eye image of the control fly. (**B**) Eye image of the dMyc overexpressed fly. (**C)** Eye of Atg1 knockdown fly. (**D**) Eye of Atg8a knockdown fly. (E) Eye of dMyc overexpression and Atg1 knockdown fly. (**F**) Eye of dMyc overexpression and Atg8a knockdown fly. (**G**) Flies expressing CTG270 repeats exhibited reduced eye size and loss of pigmentation (indicated by arrow). (**H**) dMyc overexpression led to significant improvements in eye size and pigmentation. (**I**) Knockdown of Atg1 in the disease background improved the eye phenotype. (**J**) Simultaneous knockdown of Atg1 and overexpression of dMyc significantly improved the eye phenotype. (**K**) Knockdown of Atg8a in the disease background improved the eye phenotype. (**L**) Simultaneous knockdown of Atg8a and overexpression of dMyc significantly improved the eye phenotype. (**M**) Bar graph comparing eye size between different genotypes. (**N**) Bar graph comparing the phenotypic score between different genotypes using Flynotyper (n=10 flies/genotype). All bar graphs were presented as mean ± SD and analyzed using one-way ANOVA with Tukey’s post-hoc analysis across genotypes. Statistical significance was indicated as follows: **P* < 0.05, ***P* < 0.01, *****P* < 0.0001. Scale bar (**A-L**): 100 µm.

**Video 1.** Crawling video of the control larvae.

**Video 2.** Crawling video of the dMyc overexpressed larvae.

**Video 3.** Crawling video of the disease larvae. The crawling ability of the diseased larvae was significantly reduced.

**Video 4.** Crawling video of the rescue larvae. The crawling ability was improved in the dMyc overexpressed disease larvae.

**Video 5.** Video showing improvement of the climbing ability in the rescue flies. There was a significant improvement in the climbing ability of the rescue flies.
