# Supplementary material for: dMyc suppresses CTG-induced cytotoxicity in the Drosophila model of DM1 by reducing autophagy and cell death
Source: Cell Death Discov. 2026 Apr 20;12:261. doi: 10.1038/s41420-026-03123-w (PMC13223253; doi:10.1038/s41420-026-03123-w)
Supplement: Supplementary file 7 — Original Western Blots [file 41420_2026_3123_MOESM7_ESM.docx]

**Original Western Blots**

**Fig. 2Q	Actin Blots**

**Repeat 1	Repeat 2	Repeat 3**

**Actin**

**Tubulin**

**Fig. 2S: Myosin Blots**

**Repeat 1	Repeat 2	Repeat 3**

**Myosin**

**Tubulin**

**Fig. 3F	dMyc Blots**

**Repeat 1	Repeat 2	Repeat 3**

**dMyc**

**Tubulin**

**Fig. 4P	Lamp1 Blots**

**Repeat 1	Repeat 2	Repeat 3**

**Lamp 1**

**Tubulin**

**Fig. 4R	Ref (2)P Blots**

**Repeat 1	Repeat 2	Repeat 3**

**Ref (2)P**

**Tubulin**

**Fig. 5U	Dcp1 Blots**

**Repeat 1	Repeat 2	Repeat 3**

**Dcp 1**

**Tubulin**

**Fig. 6T	Lamp 1 Blots**

**Lamp 1	Tubulin**

**Repeat 1**

**Repeat 2**

**Repeat 3**
